# The nature of correlation perception in scatterplots

**DOI:** 10.3758/s13423-016-1174-7

**Published:** 2016-10-26

**Authors:** Ronald A. Rensink

**Affiliations:** Departments of Computer Science and Psychology, University of British Columbia, Vancouver, BC Canada

**Keywords:** Perceptual organization, Visual perception, Perceptual categorization and identification, Information visualization

## Abstract

For scatterplots with gaussian distributions of dots, the perception of Pearson correlation *r* can be described by two simple laws: a linear one for discrimination, and a logarithmic one for perceived magnitude (Rensink & Baldridge, 2010). The underlying perceptual mechanisms, however, remain poorly understood. To cast light on these, four different distributions of datapoints were examined. The first had 100 points with equal variance in both dimensions. Consistent with earlier results, just noticeable difference (JND) was a linear function of the distance away from *r* = 1, and the magnitude of perceived correlation a logarithmic function of this quantity. In addition, these laws were linked, with the intercept of the JND line being the inverse of the bias in perceived magnitude. Three other conditions were also examined: a dot cloud with 25 points, a horizontal compression of the cloud, and a cloud with a uniform distribution of dots. Performance was found to be similar in all conditions. The generality and form of these laws suggest that what underlies correlation perception is not a geometric structure such as the shape of the dot cloud, but the shape of the *probability distribution* of the dots, likely inferred via a form of ensemble coding. It is suggested that this reflects the ability of observers to perceive the *information entropy* in an image, with this quantity used as a proxy for Pearson correlation.

## Introduction

The analysis of data is important in many aspects of life. An important part of such analysis is the use of graphical representations, which can be highly effective when datasets are large, messy, and complex (see e.g., Card, Mackinlay, & Shneiderman, 1999; Thomas & Cook, 2005). If a graphical representation is designed well, analysis can be rapid, accurate, and precise; in such situations the visual system of the analyst perceives structure in a dataset in much the same way as it perceives structure in the physical world. The perception of such graphical representations therefore has considerable potential to help us investigate various aspects of our visual intelligence (Rensink, 2014; see also Cleveland & McGill, 1987; Meyer, Taieb, & Flascher, 1997).

It has been argued (Rensink & Baldridge, 2010) that a good test-bed for this approach is the estimation of Pearson correlation *r* in scatterplots. In part, this is because much of the estimation of *r* appears to be a perceptual process, one to which existing techniques of vision science can be readily applied (e.g., Doherty, Anderson, Angott, & Klopfer, 2007; Meyer & Shinar, 1992; Meyer et al., 1997). Another reason is that this domain is simple enough to explore systematically, while still being rich enough to raise interesting questions about the mechanisms involved.

Historically, the perception of correlation has been investigated in several ways (for reviews, see Boynton, 2000; Doherty et al., 2007). Most were based on *numerical estimation*—asking observers for a number that describes the magnitude of the correlation perceived. Results showed that perceived correlation *g*(*r*) tends to underestimate physical correlation *r* (especially at intermediate levels), with little correlation perceived when | r | < 0.2 (Bobko & Karren, 1979; Boynton, 2000; Cleveland, Diaconis, & McGill, 1982; Strahan & Hansen, 1978). They also showed that much of this process is carried out rapidly, with results largely independent of the statistical expertise of the observer (Lane, Anderson, & Kellam, 1985; Meyer & Shinar, 1992; Meyer et al., 1997; Strahan & Hansen, 1978); indeed, particular neural systems appear to be involved (Best, Hunter, & Stewart, 2006). Thus, although knowledge and expertise can influence the more sophisticated aspects of this process (Freedman & Smith, 1996; Lewandowsky & Spence, 1989), there nevertheless seems to exist a distinct *basic stage* of correlation perception—a rapidly-acting initial phase that can be considered purely perceptual, with similar characteristics for most observers.

However, although these studies were important, they had limitations. First, they paid relatively little attention to the *precision* of the process—the extent to which the same estimate results when the same stimulus is presented. Next, the *central assumption* of the estimation techniques—that numbers can be assigned to perceived magnitudes in a consistent way—is problematic, leading to the possibility of unstable or context-dependent estimates (Ellermeier & Faulhammer, 2000) (this may help explain why inconsistent results are sometimes encountered—see e.g., Doherty et al., 2007.) Third, there was little investigation of *systematicity*—the extent to which connections exist between precision and accuracy, not to mention with the rest of visual perception. Finally, little distinction was usually made between the perception of *population* properties (e.g., the correlation of a set of scatterplots) and *particular* ones (e.g., the distance of an outlier from the dot cloud of an individual scatterplot).

Rensink and Baldridge (2010) developed an approach that took many of these considerations into account. First, precision was measured via the just noticeable difference (JND—also referred to as the *difference threshold*), the difference needed to correctly discriminate two correlations 75 % of the time. Second, accuracy was measured via *bisection*: adjusting a test plot to have its perceived correlation be halfway between those of two references. Because the visual system is more concerned with relative than absolute quantities, bisection estimates are potentially more reliable and less affected by context (Tommasi, 2000; Zimmer & Ellermeier, 2006). Next, results were analyzed for possible relationships between discrimination and perceived magnitude. Finally, to ensure that the properties of populations were being perceived, dozens of scatterplots were shown for each measurement, with population parameters held constant.

Applying this approach to scatterplots with gaussian distributions, Rensink and Baldridge (2010) found that discrimination could be described by

1 $$ \mathrm{J}\mathrm{N}\mathrm{D}(r)=k\left(1/{b}_{\mathrm{disc}}-r\right) $$

where the *variability parameter k* and *bias* (or *offset*) *b*_disc_ are such that 0 < *b*_disc_, *k* < 1. Multiplying Eq. (1) by *b*_disc_ and letting *u* = *1*- *b*_disc_ *r*, this becomes JND(*u*) = *ku*, an instance of *Weber*’*s Law*, which characterizes discriminability for many simple properties, such as length and brightness (Billock & Tsou, 2011; Ross, 1997). Meanwhile, the estimation of perceived correlation *g*(*r*) could be described by

2 $$ g(r) = \ln \left(1\ \hbox{--} {b}_{\mathrm{est}}r\right)/ \ln \left(1\ \hbox{--} {b}_{\mathrm{est}}\right) $$

where *b*_est_ is a bias parameter describing the degree of underestimation encountered. Again letting *u* = *1*- *b*_est_ *r*, this becomes *g* = ln(*u*)/(ln(1 - *b*_est_)). This is an instance of *Fechner*’*s Law*, which has been proposed for the relation between the perceived and physical magnitudes of various properties (see Ross, 1997). In addition, Rensink and Baldridge (2010) found that *b*_disc_ = *b*_est_, connecting discrimination and estimation in a systematic way.

Although these results led to a better understanding of behavior, they did not lead to a better understanding of the mechanisms responsible. To cast light on these, this study examined the generality of these results for different distributions of data points. Rensink and Baldridge (2010) used dot clouds with 100 points, with gaussian distributions of equal variance in both dimensions. To determine if any of these factors affect performance, four conditions were examined. The first replicated Rensink and Baldridge (2010): scatterplots had 100 points, with gaussian distributions of equal variance in both dimensions. A second condition tested the effects of dot cloud density, using only 25 dots. A third tested sensitivity to the shape of the dot cloud: this once again had 100 points, but was now compressed horizontally by a factor of 2. Finally, the fourth condition was similar to the first, but with a uniform instead of a gaussian distribution.

Results show that the laws found in Rensink and Baldridge (2010) are much the same for all these distributions. To account for the shape of these laws, it is suggested that correlation perception is based on the width of the probability distribution inferred from the points in the dot cloud. And to explain why perception of this structure might be useful, it is suggested that it reflects the perception of the information entropy in an image. Among other things, these results lead to a straightforward way to evaluate the effectiveness of a scatterplot design, as well as several predictions about the perception of correlation under various conditions. And at the most general level, they support the proposal that the study of the graphical representations used in information visualization can provide considerable insight into various aspects of the human visual system.

## General methods

The experimental design here was similar to that of Rensink and Baldridge (2010). Each observer was shown a set of scatterplots containing data from a set of pseudo-random numbers with a fixed mean and standard deviation in each dimension. For discrimination, observers were asked to determine which of two side-by-side scatterplots was more correlated; for perceived magnitude, a test plot was adjusted until its perceived correlation was halfway between those of two reference plots. All observers carried out both tasks, the order of which was counterbalanced. Observers were told that accuracy was important, and that they could take as much time as needed. To familiarize each observer with the discrimination task, a set of eight practice trials—easy versions of the main task—were given (with feedback) beforehand, each set continuing until the observer reached 75 % accuracy, or 32 trials had been run. For the bisection task, observers were given seven practice trials; owing to the nature of the task, no feedback could be given for these.

### Observers

Each condition had 20 observers. All were undergraduates at the University of British Columbia, and were paid $10 for a single one-hour session. All had normal or corrected-to-normal vision. Although not a requirement, all had at least some experience with scatterplots. Observers were replaced if their results (either *k* or *b*) were more than 2.5 standard deviations beyond the average of the others; based on this criterion, 2–3 observers were replaced in each experiment.^1^

### Stimuli

In all tests, observers were seated 45 cm from a screen 32° × 22° in extent. Vertical and horizontal axes of each scatterplot extended 6.3°; no tick marks or labels were used. For all conditions (except those of Experiment 3), dot clouds extended 6.3° × 6.3° and were centered on the midpoints of the axes; standard deviation was 0.2 of the extent of the cloud. And in all conditions (except those of Experiment 2), they contained 100 dots, each with a diameter of 4 min of arc (0.067°), ten times the visibility limit of 0.4 min of arc (Li, van Wijk, & Martens 2009).

For gaussian distributions, the *x*-coordinates of each dot were selected first, with this set scaled to match the given mean and standard deviation. A set of *y*-values was similarly created. Each point (*x*,*y*) was then transformed to yield the correlated pair (*x*,*y*’) via

3 $$ {y}^{\prime }=\frac{\lambda x+\left(1-\lambda \right)y}{\sqrt{\lambda^2+{\left(1-\lambda \right)}^2}},\ \mathrm{where}\ \lambda =\frac{r^2-\sqrt{r^2-{r}^4}}{2{r}^2-1}, $$

where *r* is the target correlation. To prevent values from exceeding the range of the scatterplot, any point greater than 2.5 standard deviations from the mean was eliminated, and a new point generated to take its place. Points were adjusted so that correlation was within 0.005 of the target. The resulting set was then rescaled again to have the designated mean and standard deviation.

### Procedure – discrimination

An important aspect of performance is *precision*—the scatter in the estimates made by an observer repeatedly given the same data. Following Rensink and Baldridge (2010), this was assessed via the just noticeable difference (JND), the value of ∆ for which scatterplots containing correlations *r* and *r* ± ∆ can on average be discriminated 75 % of the time. Precision and JND are directly related: the greater the JND, the greater the separation needed to see that two scatterplots have different correlations, and consequently, the worse the precision of the perceptual estimates.

**Fig. 1:**
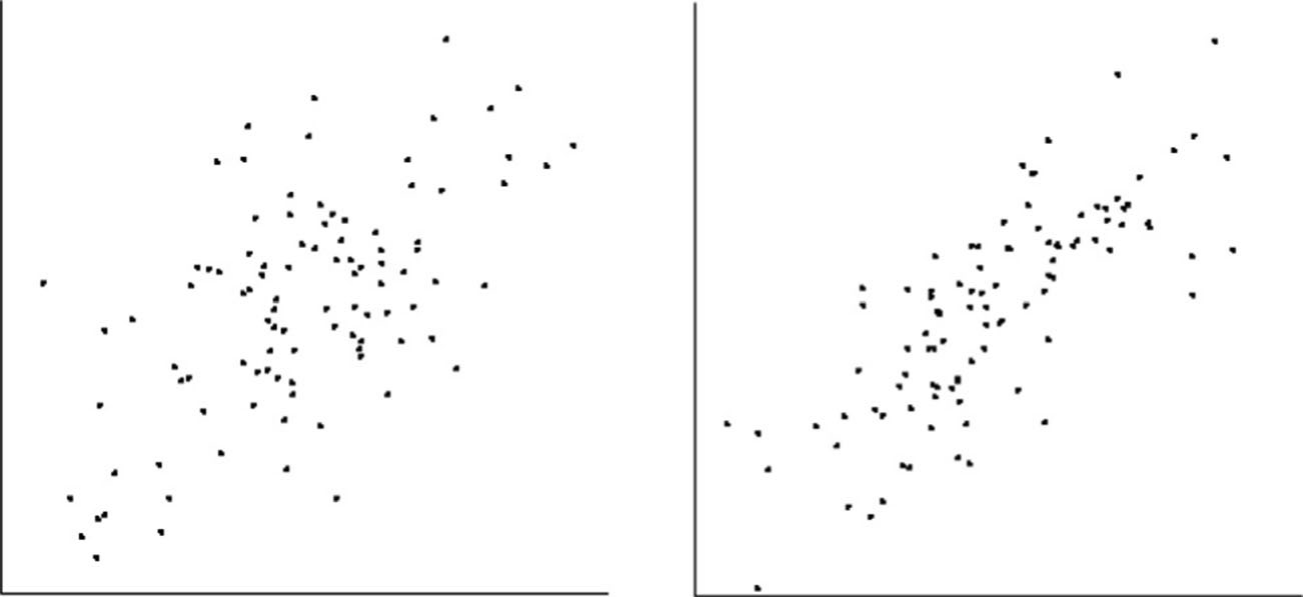
Example of discrimination task. Two side-by-side scatterplots were shown to each observer. Observers are asked to choose the one that appeared more correlated. Plots were 6.3° × 6.3° in extent, with axes of length 6.3°; dot cloud centers were separated by 15°. In this example, base correlation is 0.6 (left), and JND is from above (i.e., correlation of test plot is 0.8, higher than base correlation)

The procedure to assess JND was based on that of Rensink and Baldridge (2010). The set of correlations tested (the *base correlations*) ranged from 0.0 to 0.9, in increments of 0.1. For each, JND was obtained via a series (or *run*) of trials. In each trial, observers were shown two side-by-side scatterplots—one with the base correlation and the other a test correlation above or below it—and asked to select the more highly correlated one (Fig. 1). For each run, the initial correlation of the test plot was 0.1 above the base (when testing from above) or 0.1 below (when testing from below).^2^ If the answer was correct, the size of the difference was decreased by 0.01, making the task more difficult; if incorrect, it was increased by 0.03, making the task easier. (The three steps for each correct answer matched a single step for each incorrect answer, resulting in a steady-state accuracy of 75 %.) Following Rensink and Baldridge (2010), performance was measured via a moving window of 24 consecutive trials, divided into three sub-windows of eight trials each. After an initial 24 trials, the ratio of the variance between sub-windows to the average variance within the sub-windows (somewhat akin to an F-test)^3^ was continually calculated. Testing halted when this ratio became sufficiently low (≤0.25), or—consistent with the recommendations of Treutwein (1995)—52 trials had been run; the average of the sub-windows was then taken as the JND. This procedure proved reasonably effective, yielding results in 40 trials on average, and failing to converge on only 27 % of the runs.^4^

To assist convergence, test correlations were limited to values between the base and 1.0 (for above), or the base and 0.0 (for below). Feedback was provided immediately after each response via a 1-s sign: "+" for correct, and "−" for incorrect. New plots were generated every time a response was made, to encourage observers to respond to average properties (e.g., correlation) and avoid features of particular scatterplots (e.g., outliers).

Order of testing was determined via a latin square design (Kirk, 1995) that counterbalanced base correlations and direction of JND (above vs. below) across the 20 observers; the location of the scatterplot with the base correlation (left vs. right) was randomly assigned in each trial. To avoid floor effects at low correlations, no tests from below were run for base correlations of *r* < 0.3.

### Procedure – magnitude estimation

Another important aspect of performance is *accuracy*—the extent to which an observer can on average correctly determine the correlation of a scatterplot. Being a measure of central tendency, accuracy is, in principle, unrelated to precision.

**Fig. 2:**
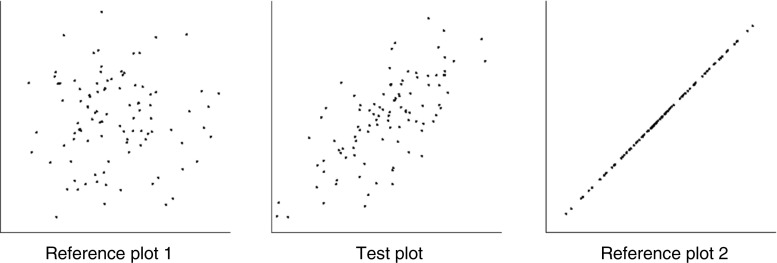
Example of magnitude estimation task. Observers adjusted the correlation of the test plot until its correlation appeared to be halfway between those of the reference plots. Plots were 6.3° × 6.3°, axes 6.3° long, with dot cloud centers separated by 7.6°. This example is taken from the initial round of bisection; the reference plots have correlations *r* = 1 and *r* = 0, while the test plot has the correlation *r* = 0.7

Following Rensink and Baldridge (2010), accuracy was measured via the bisection of perceived correlation. Here, observers were shown two horizontally-separated *reference plots* (one with a high level of correlation, one with a low) and a *test plot* placed between them (Fig. 2). The correlation of the test plot was initially 0.1 away from that of the upper or the lower reference plot (each being equally likely); the observer then adjusted it until its correlation appeared to be halfway between those of the references. This was done via keyboard control, with observers free to adjust the correlation of the test plot however they wished. To remove the possibility that observers could somehow use the number of steps, the size of each step was randomly assigned a value between 0 and 1/10 the difference between the reference correlations. Differing from Rensink and Baldridge (2010), each scatterplot—both reference and test—was replaced by a new instance every time an adjustment was made, or 1 s had passed. As in the case of discrimination, this encouraged observers to base their judgments on average properties rather than on some feature of a particular scatterplot.

The initial round of each test began with observers determining the point subjectively halfway between *r* = 0 and *r* = 1 (these corresponded to *g* = 0 and *g* = 1, respectively). This was done four times, with the mean value taken as the physical correlation *r* corresponding to perceived correlation *g* = 1/2. A second round applied this recursively on two different subconditions: in the first, observers estimated the point between *g* = 0 and 1/2, and in the second, the point between *g* = 1/2 and 1; the order of these was counterbalanced. These measurements were again made four times for each subcondition, with their averages taken as the values corresponding to *g* = 1/4 and *g* = 3/4, respectively. A third round then measured the values of *r* corresponding to perceived magnitudes *g* = 1/8, 3/8, 5/8, and 7/8; subconditions here were presented in random order.

### Analysis

Following Kay and Heer (2015), JNDs were log-transformed before calculating average values across observers. JND curves were obtained by plotting these averages against adjusted correlation *r*_A_, a symmetric measure that equates results from above and below base correlation; this is defined as *r*_A_ = *r* + 0.5 JND(*r*), the average of the two scatterplot correlations (Rensink & Baldridge, 2010). Variability *k* was the negative of the slope of this line, and bias *b*_disc_, the reciprocal of its intercept with the *r*-axis (= *k* times the reciprocal of the intercept). Individual variabilities and biases were similarly calculated from individual JND curves. Bias *b* had considerable skew and kurtosis, which was reduced by use of a probit transform, with *b* limited to ≤ .99. Also, to avoid the possibility that the limits of *r* = 0 and *r* = 1 were affecting JND estimates (Harrison, Yang, Franconeri, & Chang, 2014; Kay & Heer, 2015), a *range constraint* was imposed: a JND estimate was dropped if its average ± 2.5 standard deviations exceeded either of these limits.

In accord with the recommendations of Cumming (2012), effect sizes are emphasized in all analyses; 95 % confidence intervals (CIs; shown in square brackets) are given for all quantities of interest. Unless specified otherwise, any comparison is based on paired two-tailed *t*-tests.

## Experiment 1 – basic condition

**Fig. 3:**
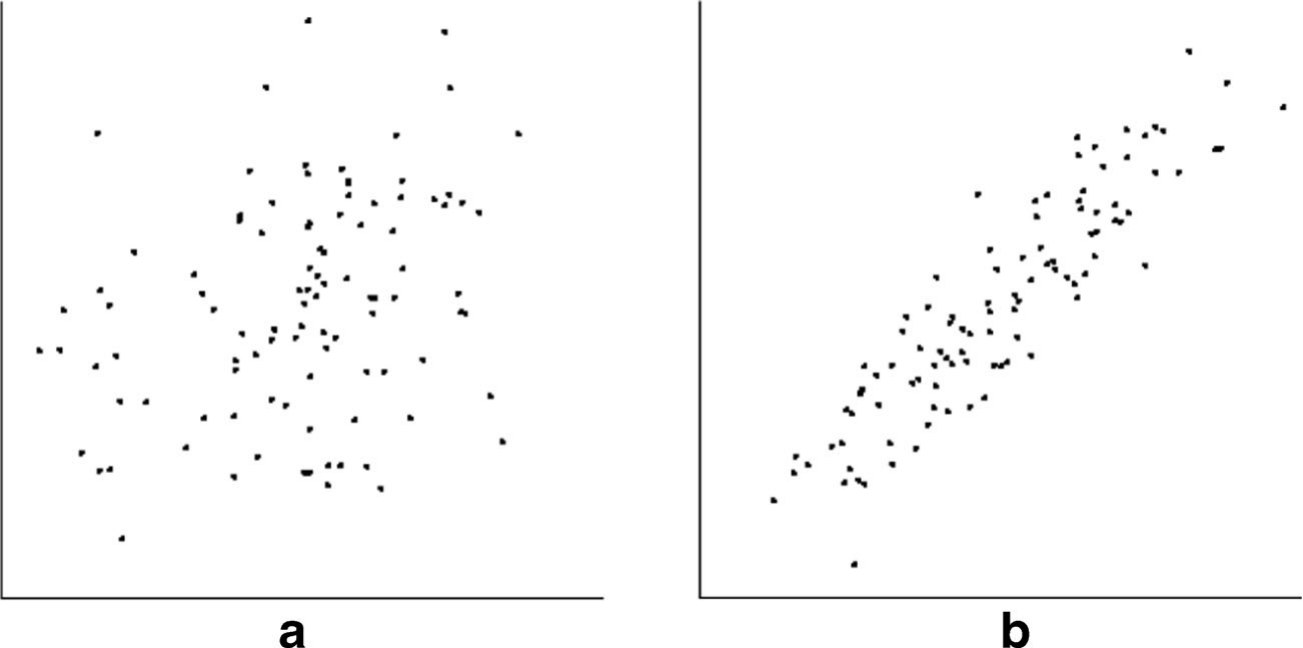
Examples of scatterplots for the basic condition. Here, scatterplots had 100 points with the same gaussian distribution in both dimensions. (**a**) Typical scatterplot for *r* = 0.3, (**b**) typical scatterplot for *r* = 0.9

The goal of this experiment was to replicate Rensink and Baldridge (2010), and serve as a "basic" condition against which the others could be compared. Dot clouds had 100 points, in a gaussian distribution with a mean of 0.5 and standard deviation of 0.2 in both dimensions (Fig. 3).

### Results: Discrimination

**Fig. 4:**
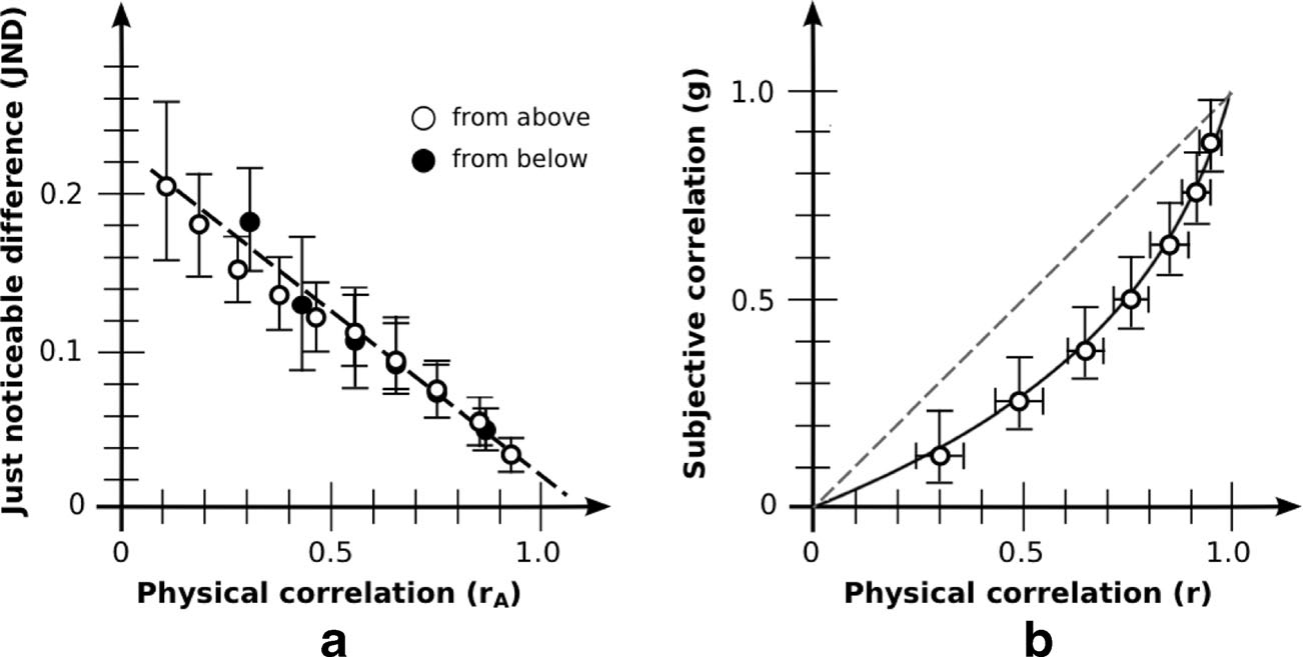
Aggregate results: basic condition. (**a**) Discrimination as measured via JND. Error bars denote 95 % CIs. White dots indicate that comparison is made against a test correlation from above; black dots indicate test correlation from below. As is evident, these give rise to much the same line. Note that the maximum error in *r* in the generated plots (of 0.005) is much less than the JNDs found, even those for high correlations, making it unlikely that this affects estimates in any significant way. (**b**) Magnitude estimation as measured via bisection. The curve for perceived correlation is g(*r*) = ln(1– *b*_est_r)/ln(1– *b*_est_); best fit is for *b*_est_ = 0.90. Vertical error bars show g(r ± 1 JND); horizontal error bars the 95 % CIs. The reference line g(*r*) = *r* makes explicit the degree of underestimation from physical correlation

JNDs based on aggregate data are shown in Fig. 4a. Due to the range constraint, JNDs for base correlation *r*_B_ = 0.3 (from below) were omitted from the analysis. Similar to what was found in Rensink and Baldridge (2010), JND was a strongly linear function of adjusted correlation both for JNDs from above (*R* ^2^ = .987) and below (*R* ^2^ = .941). For each observer, slopes and intercepts with the *y*-axis were then obtained by fitting a least-squares line through their data points. Consistent with Rensink and Baldridge (2010), there was no effect of JND direction on either slope (−0.20 [−0.15, −0.24] for above; −0.23 [−0.18, −0.28] for below; *t*(19) = 1.12; *p* = .28) or intercept (0.23 [0.19, 0.26] for above; 0.25 [0.21, 0.29] for below; *t*(19) = 1.14; *p* = .27). Combining the data for both directions, JND retained a strong linearity (*R* ^2^ = .970). Performance is therefore well described by Eq. (1), viz.,

$$ \mathrm{J}\mathrm{N}\mathrm{D}(r)=k\left(1/{b}_{\mathrm{disc}}\hbox{--} {r}_{\mathrm{A}}\right) $$

with *k* the negative of the slope, and *b*_disc_ the reciprocal of the intercept with the *r*-axis.

Based on the variability and bias for each observer, average results were *k* = 0.21 [0.17, 0.24] and *b*_disc_ = 0.90 [0.84, 0.94]. These are broadly similar to the values *k* = 0.24 and *b*_disc_ = 0.91 of Rensink and Baldridge (2010).

### Results: Magnitude estimation

Bisection results based on aggregate data are shown in Fig. 4b. Consistent with other reports (e.g., Cleveland et al., 1982), an underestimation of correlation appeared, especially in the range 0.2 < *r* < 0.6. And consistent with Rensink and Baldridge (2010), data show a good fit to Eq. (2)

$$ g(r) = \ln \left(1\hbox{--} {b}_{\mathrm{est}}r\right)/\  \ln \left(1\hbox{--}\ {b}_{\mathrm{est}}\right) $$

where *b*_est_ is the bias obtained via magnitude estimation.

The best fit with the aggregrate data is for *b*_est_ = 0.90. The resulting fit is excellent: root mean square error (RMSE) from the set of observed values is only 0.018. The value of *b*_est_ obtained via individual estimates is similar: 0.91 [0.85, 0.95]. These results are also not far from the value *b*_est_ = 0.87 found in Rensink and Baldridge (2010).

### Results: Systematicity

If the *Fechner assumption* holds for this situation—i.e., that each JND corresponds to the same difference in subjective experience—Eq. (1) implies Eq. (2), with the bias in the two equations being identical (Rensink & Baldridge, 2010). The value of bias obtained via discrimination (*b*_disc_ = 0.90 [0.84, 0.94]) was indeed much the same as that obtained via magnitude estimation (0.91 [0.85, 0.95]); the slight difference of 0.01 was not statistically significant (*t*(19) = 0.40; *p* = .69). As such, the Fechner assumption appears to hold in this condition.

These results therefore replicate the findings of Rensink and Baldridge (2010): for this condition, average precision and accuracy of correlation perception can be described via a pair of simple performance laws (linear and logarithmic, respectively) that are closely related, with *b*_disc_ and *b*_est_ essentially measuring the same quantity *b*.

## Experiment 2 – low density

**Fig. 5:**
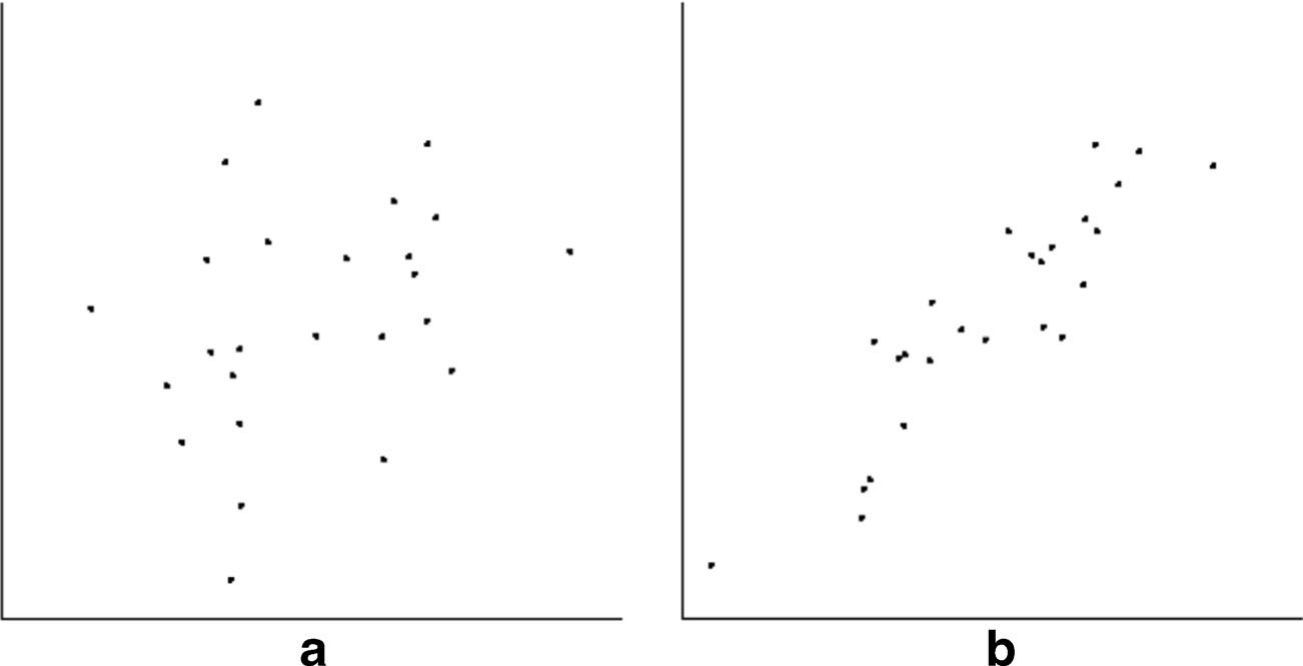
Examples of low-density scatterplots. Here, scatterplots had 25 points with the same gaussian distribution in both dimensions. (**a**) Typical scatterplot for *r* = 0.3, (**b**) typical scatterplot for *r* = 0.9

This was the same as the basic condition, but with only 25 points. The goal here was to determine whether performance would change if the number of points—and thus, the density of the dot cloud—were markedly lower (Fig. 5).

### Results: Discrimination

**Fig. 6:**
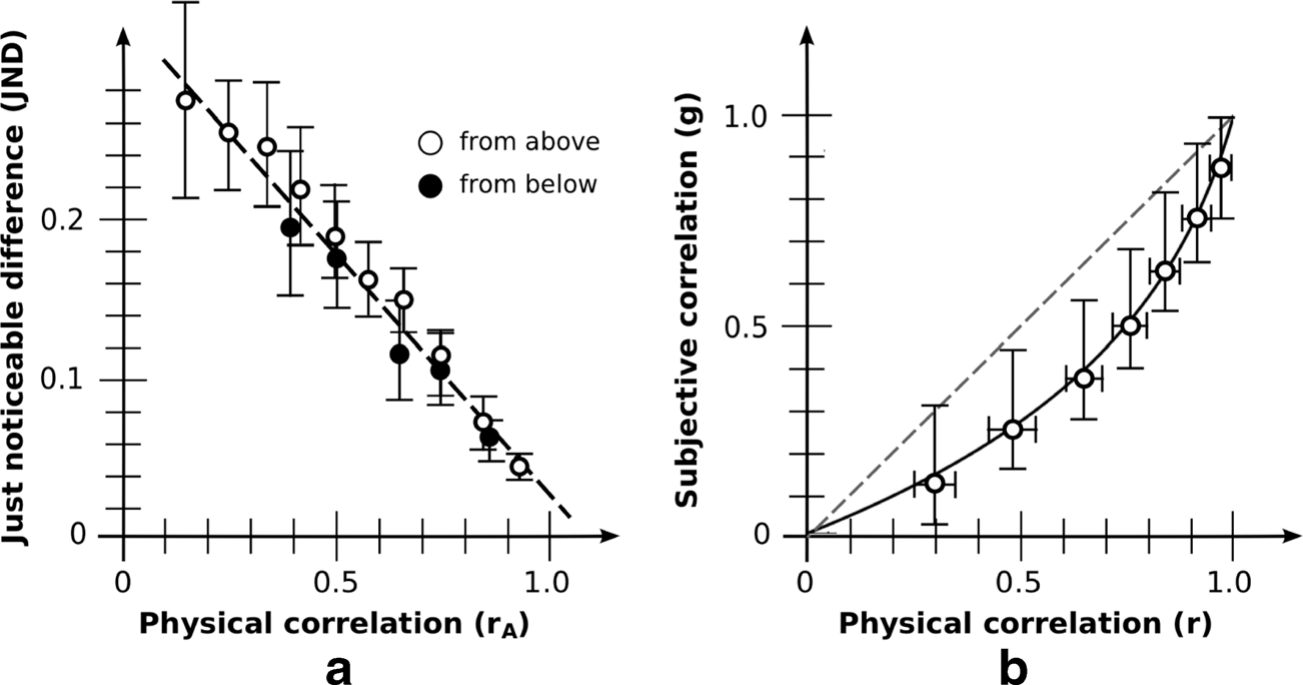
Aggregate results: low-density condition. (**a**) Discrimination as measured via JND. White dots indicate test correlation from above; black dots indicate test correlation from below. As is evident, these give much the same estimates. Error bars denote 95 % CIs. (**b**) Magnitude estimation as measured via bisection. The curve for perceived correlation is g(*r*) = ln(1– *b*_est_r)/ln(1– *b*_est_); best fit is for *b*_est_ = 0.89. Vertical error bars show g(r ± 1 JND); horizontal error bars the 95 % CIs

JNDs based on the aggregate data are shown in Fig. 6a. Due to the range constraint, results were omitted from base correlations 0.3 and 0.4 for JNDs for the below condition. The remaining JNDs were again a linear function of adjusted correlation r_A_ both from above (*R* ^2^ = .973) and from below (*R* ^2^ = .961). No effect of JND direction was found on either slope (0.30 [0.25, 0.34] for above; 0.30 [0.23, 0.36] for below; *t*(19) = 0.02; *p* = .98) or intercept (0.34 [0.31, 0.38] for above; 0.33 [0.28, 0.37] for below; *t*(19) = 0.78; *p* = .45). Combining both sets, behavior again remained quite linear (*R* ^2^ = .963).

The slopes of individual fits yielded an average variability *k* = 0.30 [0.26, 0.35], noticeably higher than for the basic condition (*k* = 0.20). Bias *b*_disc_ = 0.91 [0.85, 0.95] remained about the same (cf. *b*_disc_ = 0.90 [0.84, 0.94]).

### Results: Magnitude estimation

Estimates based on the aggregate data are shown in Fig. 6b. Data again show a good fit to the logarithmic function of Eq. (2). The best fit was for *b*_est_ = 0.89, close to the value of 0.90 for the basic condition; the fit for this against the set of observed estimates was again excellent, with an RMSE of only 0.013. The estimate of *b*_est_ obtained via individual observers was also similar: *b*_est_ = 0.90 [0.84, 0.94], and much the same as that of the basic condition (*b*_est_ = 0.91 [0.85, 0.95]).

### Results: Systematicity

The bias obtained via discrimination (*b*_disc_ = 0.91 [0.85, 0.95]) was much the same as that obtained via magnitude estimation (*b*_est_ = 0.90 [0.84, 0.94]). The difference of 0.01 was not statistically significant (*t*(19) = 0.14; *p* = .89), showing that the Fechner assumption again holds, with *b*_disc_ and *b*_est_ simply being measures of the same quantity.

## Experiment 3 – high aspect ratio

**Fig. 7:**
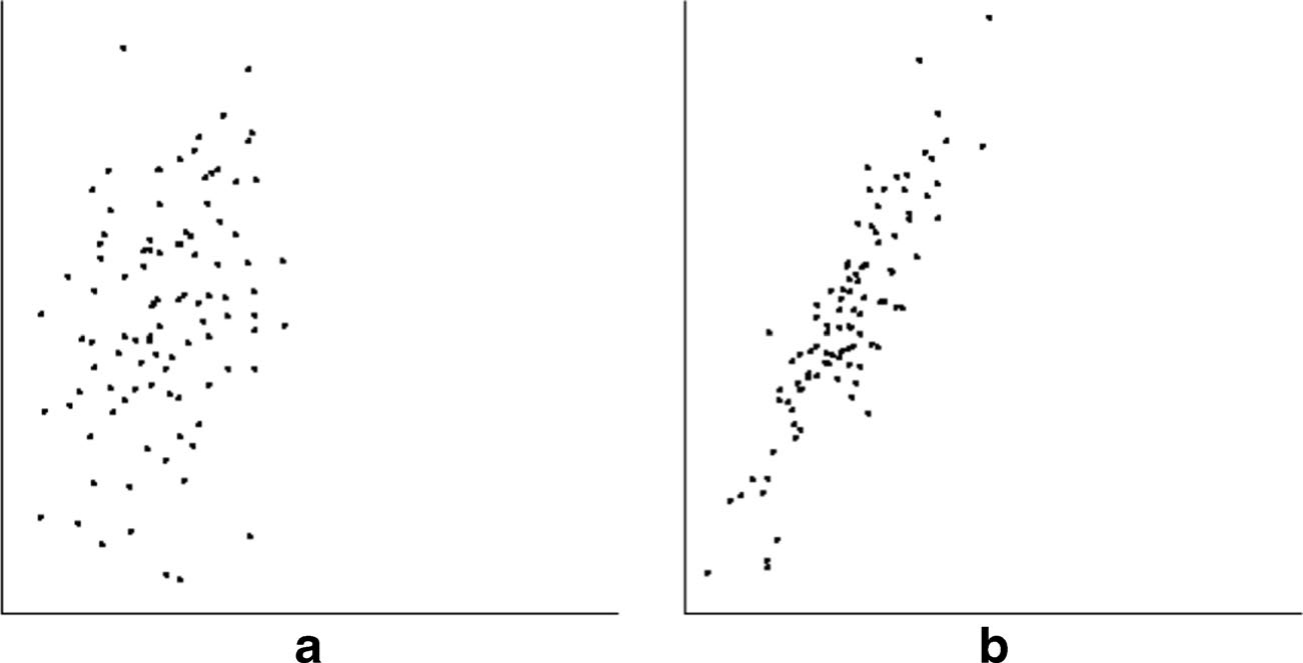
Examples of scatterplots with high aspect ratios. Here, scatterplots had 100 points with a gaussian distribution in both dimensions, but with the horizontal component compressed by a factor of 2. (**a**) Typical scatterplot for *r* = 0.3, (**b**) typical scatterplot for *r* = 0.9

An important property of any scatterplot is its *aspect ratio*—the ratio of vertical to horizontal extent. This ratio may affect the ability of observers to detect trends in data, including the perception of clusters (Fink, Haunert, Spoerhase, & Wolff, 2013), possibly by reducing the distances between the dots. To test whether perceived correlation is affected by this factor, the dot cloud of the basic condition was horizontally compressed so as to have an aspect ratio of 2:1. The size of the axes and dots themselves were left unaltered (Fig. 7).

### Results: Discrimination

**Fig. 8:**
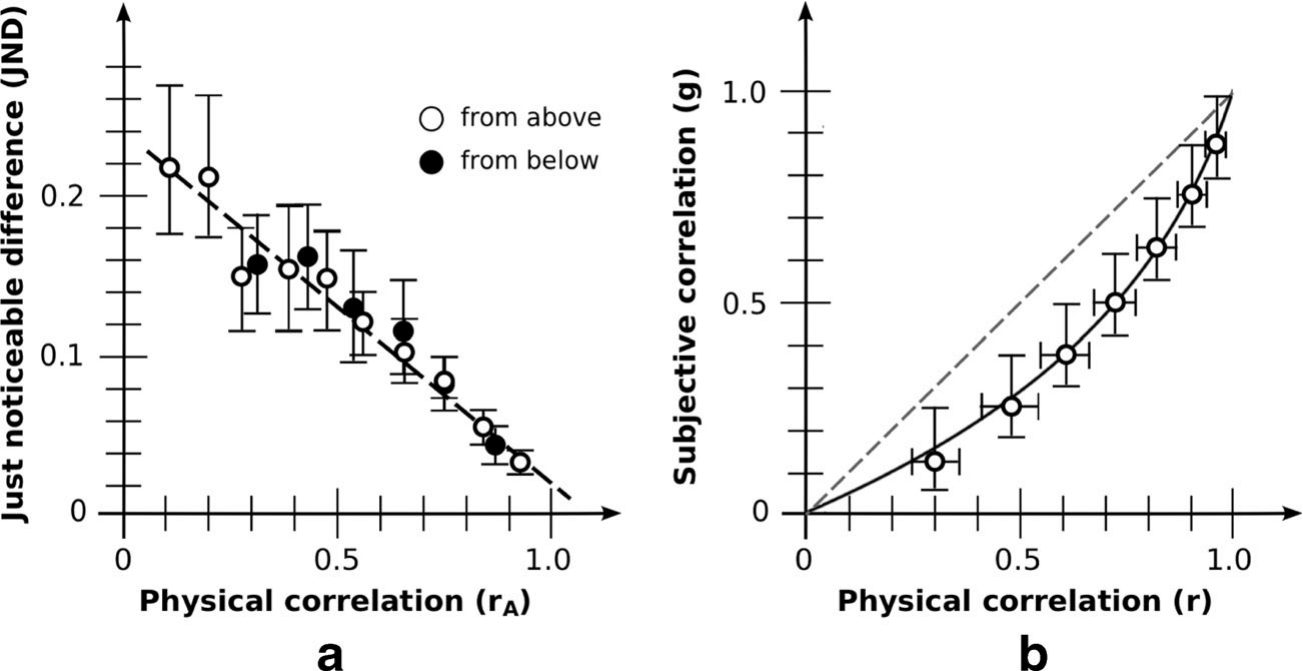
Aggregate results: high aspect ratio. (**a**) Discrimination as measured via JND. White dots indicate test correlation from above, black from below; these give much the same estimates. Error bars denote 95 % CIs. (**b**) Magnitude estimation as measured via bisection. The curve for perceived correlation is g(*r*) = ln(1– *b*_est_r)/ln(1– *b*_est_); best fit is for *b*_est_ = 0.85. Vertical error bars show g(r ± 1 JND); horizontal error bars the 95 % CIs

JNDs based on the aggregate data are shown in Fig. 8a. Results were omitted from base correlation *r*_B_ = 0.3 (JNDs from below) due to the range constraint. Dependence of JND on adjusted correlation r_A_ was again quite linear, both for JNDs from above (*R* ^2^ = .942) and below (*R* ^2^ = .942). There was no significant effect of JND direction on either slope (0.22 [0.18, 0.26] for above; 0.24 [0.18, 0.29] for below; *t*(19) = 0.57; *p* = .57) or intercept (0.25 [0.22, 0.29] for above; 0.26 [0.22, 0.31] for below; *t*(19) = 0.49; *p* = .63). When both sets of data were combined, JND remained a linear function of r_A_ (*R* ^2^ = .941).

Analysis of individual slopes yielded an average variability *k* = 0.22 [0.19, 0.26], similar to that for the basic condition (*k* = 0.21). Average bias *b*_disc_ = 0.89 [0.84, 0.92] was also much the same as the corresponding basic value (*b*_disc_ = 0.90). Thus, even when standard deviations in the two dimensions differ by a factor of 2, performance appears largely unaffected.

### Results: Magnitude estimation

Average estimates are shown in Fig. 8b. Data again show a good fit with Eq. (2). The best fit was for *b*_est_ = 0.85; the resulting fit is excellent, with RMSE less than 0.013. The average value of *b*_est_ obtained via individual observers was similar: *b*_est_ = 0.83 [0.71, 0.92].

### Results: Systematicity

For this condition, the bias obtained via discrimination (*b*_disc_ = 0.89 [0.84, 0.92]) was not far from that obtained via magnitude estimation (*b*_est_ = 0.83 [0.71, 0.92]). This difference was not statistically significant (*t*(19) = 1.00; *p* = .33), suggesting that the link between precision and accuracy exists for this condition as well. Thus, for high aspect ratios, the shape of both performance curves remains much the same as for the other conditions, as does the link between them.

## Experiment 4 – uniform distribution

The results of Experiments 1–3 suggest that correlation perception is robust to variations in the parameters of a scatterplot dot cloud, at least for gaussian distributions. To examine what happens when distributions move away from being gaussian, Experiment 4 used scatterplots with uniform distributions. Such distributions have often been used to study visual perception (e.g., Chong & Treisman, 2003; Cohen, Singh, & Maloney, 2008). Moreover, they are not entirely unnatural: for example, uniform distributions appear to be the basis of internal representations of visuomotor error in speeded reaching tasks (Zhang, Daw, & Maloney, 2015).

**Fig. 9:**
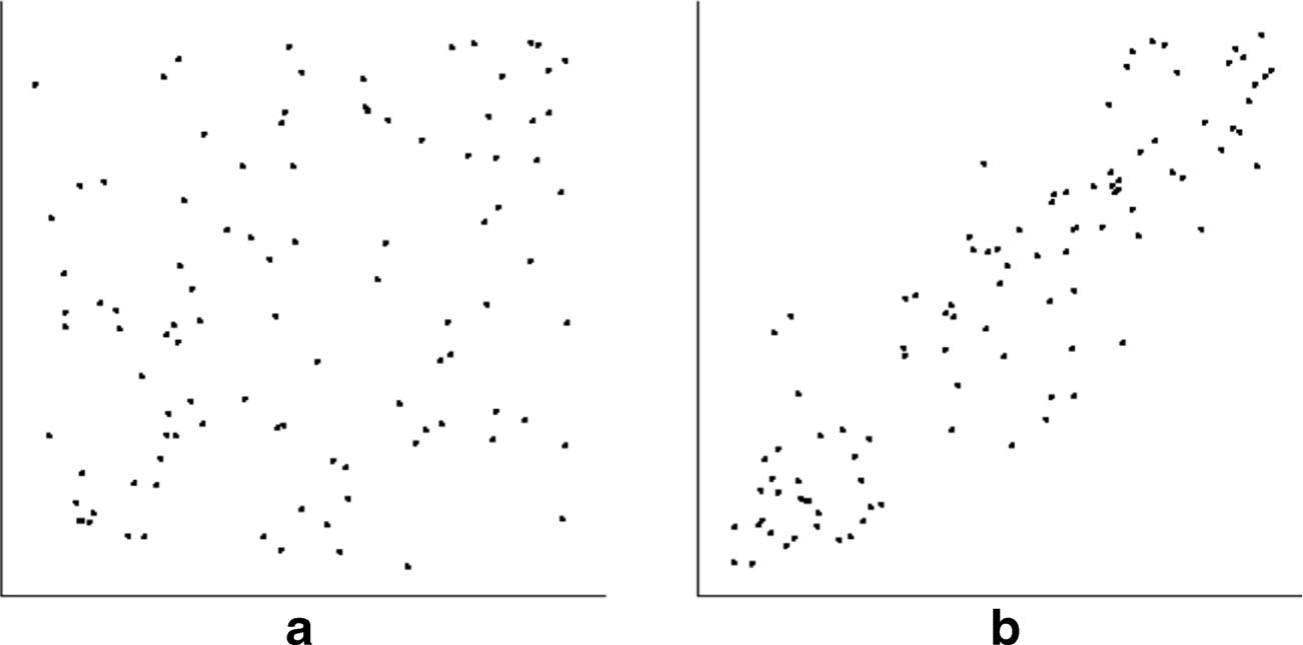
Examples of scatterplots with uniform distributions. Here, scatterplots had 100 points with a uniform distribution in both dimensions. (**a**) Typical scatterplot for *r* = 0.3, (**b**) typical scatterplot for *r* = 0.9. These tend to be slightly blockier in shape, and have more sharply-defined borders than their gaussian counterparts (cf. Fig. 3)

For the condition here, the same mean and range were used as for the basic condition; this entailed a somewhat larger standard deviation ($1/\sqrt{12}$ = 0.29, rather than 0.2). In contrast to the other conditions, points here were created by first obtaining a list of *x*-coordinates from a uniform distribution (subject to a particular mean and standard deviation). The corresponding *y*-coordinates were formed by creating a scrambled copy of this list and then ordering the values using a comb sort (Box & Lacey, 1991; Harrison, 1995) until the required correlation had been achieved. A comb sort initially compares—and if need be, swaps—items separated by large distances, with this distance being reduced over time. The result is a dot cloud with a shape somewhat similar to that of the gaussian distribution with the same correlation (Fig. 9).

### Results: Discrimination

**Fig. 10:**
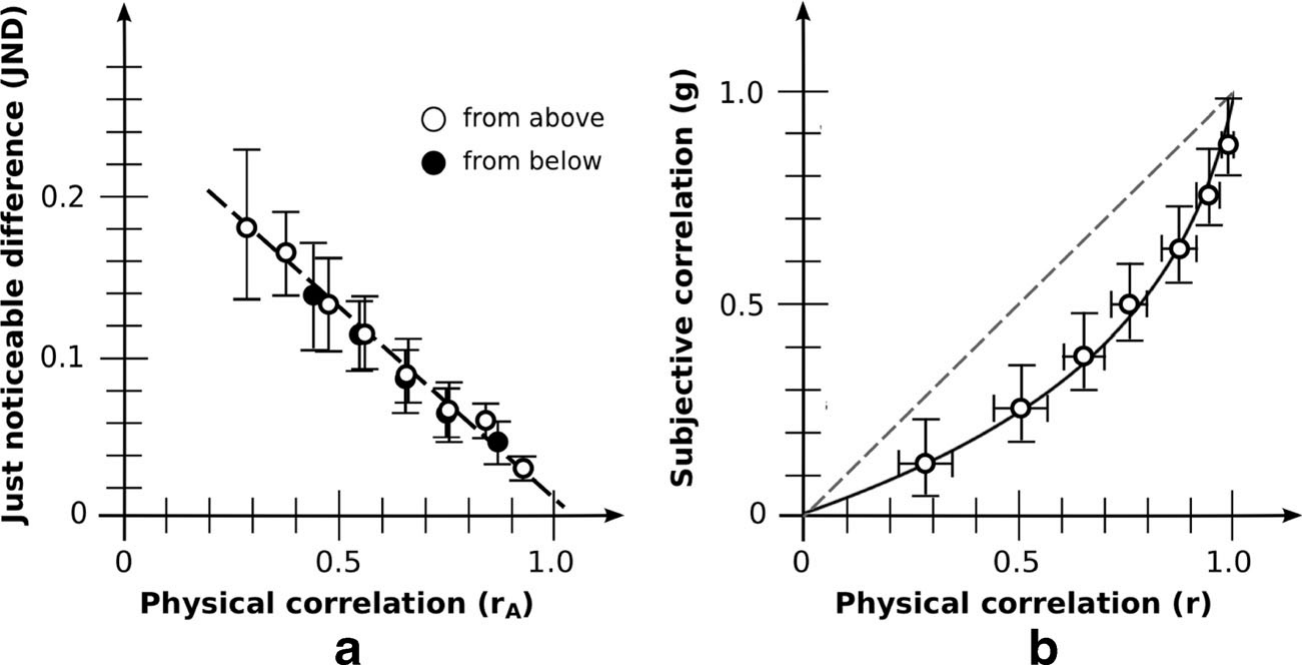
Aggregate results: uniform distribution. (**a**) Discrimination via JND. White dots indicate test correlation from above; black dots from below. As is evident, estimates are much the same. Error bars denote 95 % CIs. (**b**) Magnitude estimation as measured via bisection. The curve for perceived correlation is g(*r*) = ln(1– *b*_est_r)/ln(1– *b*_est_); best fit is for *b*_est_ = 0.93. Vertical error bars show g(r ± 1 JND); horizontal error bars the 95 % CIs

JNDs based on the aggregate data are shown in Fig. 10a. Due to the range constraint, results were omitted for base correlation *r*_B_ = 0.3 and 0.4 (JNDs from below). As in previous conditions, dependence of JND on adjusted correlation *r*_A_ was highly linear, both for JNDs from above (*R* ^2^ = .975) and below (*R* ^2^ = .988). There was a tendency for JND direction to affect slope (0.28 [0.23, 0.32] for above; 0.22 [0.16, 0.28] for below (*t*(19) = 2.09; *p* = .05).^5^ There was also an effect of JND direction on intercept (0.29 [0.25, 0.32] for above; 0.24 [0.19, 0.28] for below; *t*(19) = 2.48; *p* = .02). When these data were combined, behavior remained highly linear (*R* ^2^ = .983).

Analysis of individual slopes yielded a variability *k* = 0.24 [0.20, 0.28], not far from that of the basic condition (*k* = 0.20). Bias *b*_disc_ = 0.94 [0.89, 0.97] appeared to be slightly higher than the basic value *b*_disc_ = 0.90 [0.84, 0.94], possibly because of the greater standard deviation (Cleveland et al., 1982; Lauer & Post, 1989).

### Results: Magnitude estimation

Average estimates for this condition are shown in Fig. 10b. The best fit of the aggregate data with Eq. (2) was for *b*_est_ = 0.93. As was the case for the other conditions, the fit is a good one, with an RMSE of 0.032. This value of *b*_est_ is similar to that obtained via the fits of individual observers: 0.94 [0.91, 0.97]. Similar to the case of discrimination-based estimates, this latter value appears slightly higher than that for the basic condition (*b*_est_ = 0.91 [0.85, 0.95]).

### Results: Systematicity

As in the case of the gaussian distributions, the bias obtained via discrimination (*b*_disc_ = 0.94 [0.89, 0.97]) was much the same as that obtained via magnitude estimation (*b*_est_ = 0.94 [0.91, 0.97]). This difference was not significant (*t*(19) = 0.54; *p* = .59), indicating that the link between discrimination and magnitude estimation is reasonably good here as well.

In summary, then, performance for uniform distributions was largely the same as for gaussian ones: performance for discrimination remained highly linear, perceived magnitude remained logarithmic, and the two curves remained closely linked.

## General discussion

The experiments here show that under a fairly wide range of conditions the perception of correlation in scatterplots obeys two linked laws: a linear (Weber) law for discrimination, and a logarithmic (Fechner) law for perceived magnitude. Fit to observed values was good for all conditions tested. As such, these laws will likely hold reasonably well for many distributions, including those that are non-gaussian to some extent.

### Mechanism

Why do the laws describing correlation perception have such generality? And why do they have the form that they do? In what follows, it is suggested that (i) perceived correlation is based on the probability distribution of data points in an abstract parameter space, (ii) performance depends on the shape—and in particular, the width—of this distribution, and (iii) this reflects the ability of human observers to perceive the information entropy (Shannon entropy) in the image. Each of these suggestions will now be discussed in turn.

**Fig. 11:**
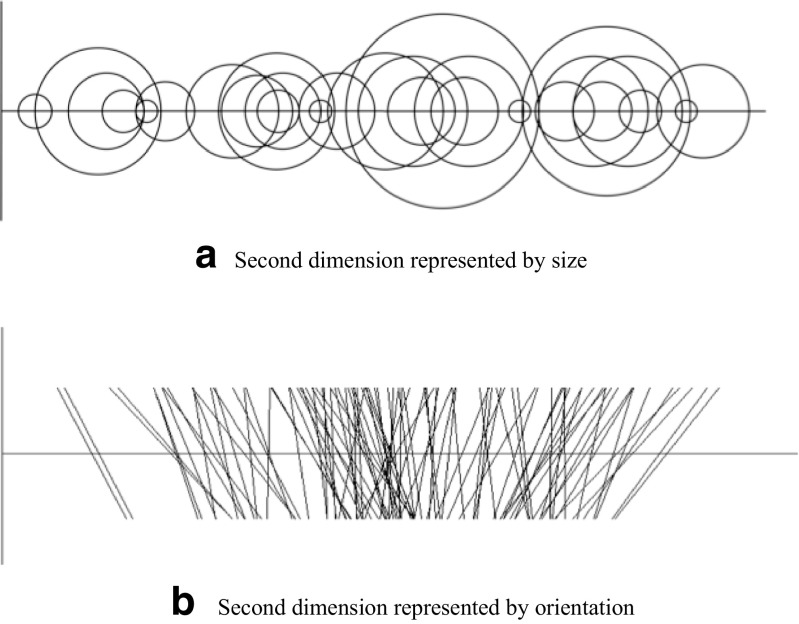
Examples of different representations of data. In these *augmented stripplots*, the first data dimension of each data element is represented spatially (as in a scatterplot), but the value of the second dimension is mapped to a value on a different physical property. (**a**) Second dimension represented by size (area). (**b**) Second dimension represented by orientation. Despite their different visual appearance, both result in correlation perception that is much the same as that for scatterplots: perceived magnitude is still described by g(*r*) = ln(1– *b*r)/ln(1– *b*), and discrimination by JND(*r*) = *k*(*1*/*b* - *r*). From Rensink (2014, 2015)
  This indifference suggests that performance is based not on a geometric structure inferred from the dot cloud, but on something more abstract. One possibility is the *probability distribution p*(*x*_i_, *y*_i_), which states that *np*(*x*_i_, *y*_i_) dots—or more generally, data points—are expected at each position *i* in a two-dimensional array. Such distributions are the basis of various scene statistics, which play an important role in scene perception (see e.g., Geisler, 2008; Olshausen & Field, 1996). Distributions involving two perceptual dimensions would not be problematic, especially if they pertain not to different properties (e.g., one for space and one for orientation), but to a common, more abstract parameter space.
  The mechanisms responsible are less clear, but may well be those that underlie ensemble coding, which enable various statistics of a set of items to be determined rapidly and with relatively little attention (see e.g., Alvarez, 2011; Haberman & Whitney, 2012). It has been suggested that the shape of one-dimensional distributions of various properties can be perceived this way (Utochkin, 2015). An extension to two dimensions would appear to be fairly natural. And given that summary statistics can be obtained in as little as 100 ms (Chong & Treisman, 2003; Robitaille & Harris, 2011), it could also explain why correlation perception can be achieved within a similarly brief amount of time (Rensink, 2014).
ii) *Distribution shape*. If the density of points is sufficiently high, *p*(*x*_i_, *y*_i_) can be approximated by a continuous *probability density function f*(*x*,*y*), such that *nf*(*x*,*y*)∆*x*∆*y* dots are expected in the area ∆*x*∆*y* centered on (*x*,*y*). The function *nf*(*x*,*y*)—the *dot density function*—has a shape largely unaffected by outliers. Moreover, the shape of *f*(*x*,*y*) does not depend on the number of dots present; the greater variability in *k* when relatively few dots are used (cf. Experiment 2; Rensink, 2014) is likely due to *f*(*x*,*y*) being sampled insufficiently finely. If the center of mass of each dot were used as the basis of *f*(*x*,*y*) (instead of raw pixels, say), it would also explain why performance is largely indifferent to their size, shape, and color (Rensink, 2014).
  For a bivariate gaussian distribution, f(x,y) has the form:
  4 $$ f\left(x,y\right)=\frac{e^{-q\left(x,y\right)}}{2\pi {\sigma}_x{\sigma}_y{\left(1-{r}^2\right)}^{1/2}} $$
  where
  5 $$ q\left(x,y\right)=\frac{\frac{{\left(x-{\mu}_x\right)}^2}{\sigma_x^2}-2r\frac{\left(x-{\mu}_x\right)\left(y-{\mu}_y\right)}{\sigma_x{\sigma}_y}+\frac{{\left(y-{\mu}_y\right)}^2}{\sigma_y^2}}{2\left(1-{r}^2\right)}, $$
  where *μ*_*i*_ and σ_i_ are respectively the mean and standard deviation of dimension *i*, and *r* is the correlation^7^ (see e.g., Timm, 2002). One way of determining its shape via a set of *isofraction* points—points whose value is a fixed fraction of the maximum (i.e., the value at the center of the dot density function). Writing this fraction as ${e}^{-{K}_1^2}$, where *K*_1_ is some fixed constant, these points form an ellipse q(x,y) = *K*_1_^2^ (Fig. 12).^8^

i) *Probability distribution*. When considering what might underlie the perception of correlation, it is worth noting that discrimination and magnitude estimation are both functions of *u* = *1*–*br*, not *r* alone. The quantity *u* is akin to the average perpendicular distance X from the regression line proposed by Meyer et al. (1997) in that it has a small value at *r* = 1 and increases as *r* decreases. The possibility that a quantity of this kind is involved is further supported by the finding that the areas of the brain involved in correlation perception increase in activity as *r* moves away from 1 (Best et al., 2006).
  One candidate consistent with this behavior is the area of the region encompassing the set of dots in the cloud—e.g., their bounding box or convex hull. This seems unlikely, however, given that outliers (e.g., from contaminating distributions) have relatively little effect on performance, which is based instead on the bulk of the points (Bobko & Karren, 1979; Konarski, 2005; Meyer & Shinar, 1992). Moreover, Weber’s Law applies best to properties that—like lightness, color, or density—are *intensive*, i.e., when components with the same property are combined, the whole has that property too (see Ross, 1997).^6^ Intensive properties that are geometric—such as orientation—are possible candidates. But those that are possible in theory do not seem likely in practice: perceived correlation corresponds neither to the orientation of the regression line (Experiment 3; Lane et al., 1985) nor to the ratio of the major and minor axes of the dot cloud (Cleveland et al., 1982; although see Boynton, 2000). Furthermore, the perceived magnitude of most physical properties (including area or distance) is generally described best by a power function of its physical magnitude (see e.g., Billock & Tsou, 2011; Ross, 1997), not a logarithmic function of the kind found here.
  An even more important consideration perhaps is the invariance of correlation perception to different kinds of graphical representation. Large dots in a scatterplot, for example, make the dot cloud blobby and give it a larger outer boundary. They do not, however, affect performance (Rensink, 2012, 2014). Indeed, estimation and discrimination of correlation follow similar laws even when the graphical representations involved are entirely different in appearance (Fig. 11)—for example, when the second data dimension of a data element is represented by size or color rather than vertical position (Rensink, 2012, 2014, 2015), or when line graphs or bar charts are used (Harrison et al., 2014).

**Fig. 12:**
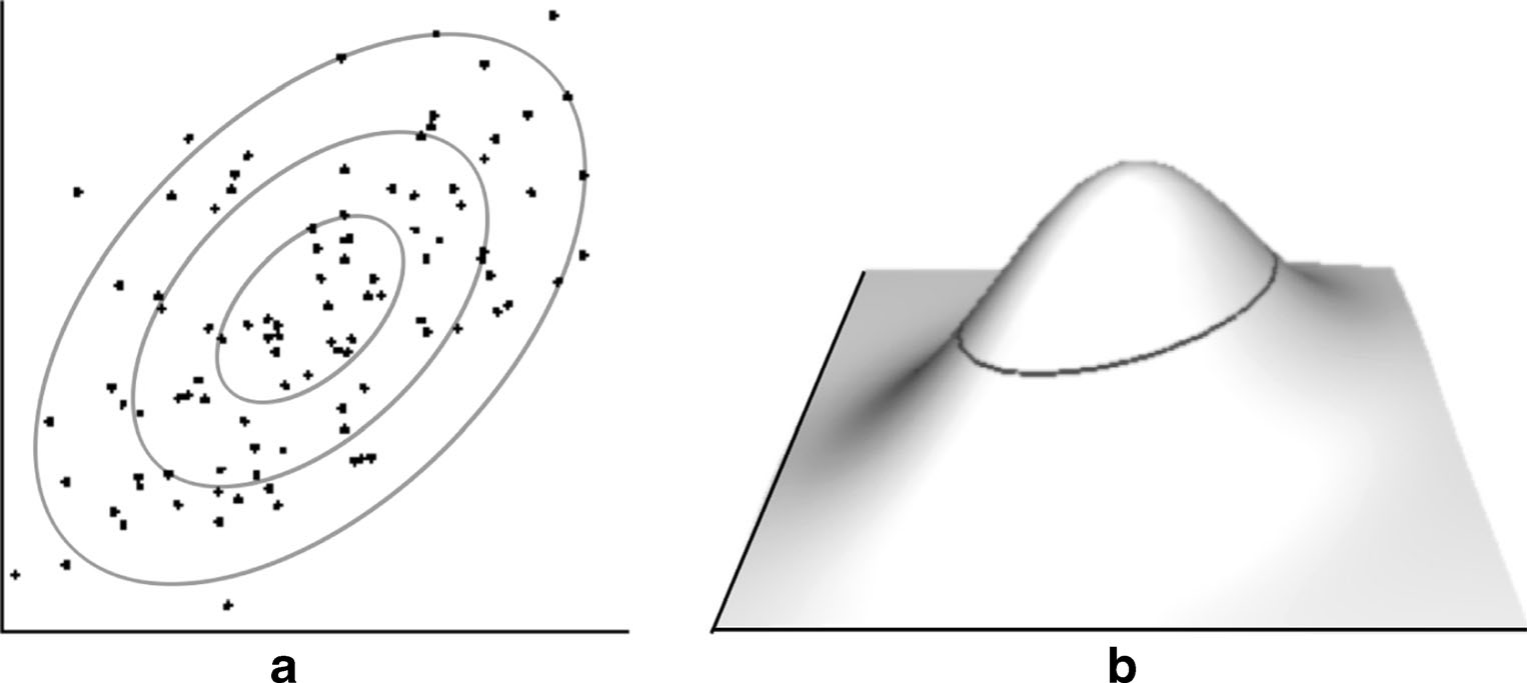
Shape of dot density function. (**a**) Two-dimensional view of a scatterplot (*r* = 0.5), with several possible isopleths (iso-probability contours). (**b**) Three-dimensional view of the corresponding dot density function *nf*(*x*,*y*), with isofraction ellipse for values 1/2 that of the maximum (i.e., the value at its center)
  Applying the formula for the semi-minor axis *L*_*min*_ of an ellipse (e.g., Johnson, 2015), the width *w* of this *isofraction ellipse* is
  6 $$ w(r)=2\times {L}_{\min }=\frac{4{K}_1{\sigma}_x\sqrt{1-{r}^2}}{{\left[\left(1+1/{\kappa}^2\right)+\sqrt{{\left(1-1/{\kappa}^2\right)}^2+4{r}^2/{\kappa}^2}\right]}^{1/2}}, $$
  where *κ* = *σ*_*y*_/*σ*_*x*_. Note that *w*(*r*) is independent of *n*. Given Eq. (2), it is natural to assume that perceived correlation *g*(*r*) might be proportional to the logarithm of this quantity. This assumption can be expressed as
  7 $$ g(r)=G\left[ \ln \left(w(r)\right)+h\right]. $$
  where *G* and *h* are constants, to be chosen such that *g*(0) = 0 and *g*(1) = 1. Note that this implies a calibration step to map perceived quantities to the appropriate values of correlation; skipping this step may explain some of the incoherence in magnitude estimates occasionally encountered (see e.g., Doherty et al., 2007).
  When both dimensions have the same standard deviation σ, Eq. (6) reduces to
  8 $$ w(r)=2\sqrt{2}{K}_1\sigma \sqrt{1-r}, $$
  and Eq. (7) takes the form
  9 $$ g(r)=\frac{G}{2} \ln \left({K}_2^2\left(1-r\right)\right)+Gh $$
  where ${K}_2=2\sqrt{2}{K}_1\sigma$. Note that *g*(*r*) diverges as *r* approaches 1, since the width of the ellipse approaches zero. However, owing to perceptual noise and blurring, a residual width *w*_*res*_ still exists in this situation. Placing this into Eq. (9) yields
  10 $$ g(r)=\frac{G}{2} \ln \left({w}_{res}^2+{K}_2^2\left(1-r\right)\right)+Gh, $$
  which can be rewritten
  11 $$ g(r)=\frac{G}{2}\left[ \ln \left(1-br\right)+ \ln \left({w}_{res}^2+{K}_2^2\right)\right]+Gh, $$
  where
  12 $$ b=\frac{K_2^2}{w_{res}^2+{K}_2^2}. $$
  Choosing *h* such that *g*(0) = 0, Eq. (11) becomes
  $$ g(r)=\frac{G}{2} \ln \left(1-br\right), $$
  which becomes Eq. (2) when *G* is chosen to let *g*(1) = 1.
  In this view, the parameter *b* reflects the relative contribution of residual and "primary" factors. This can be made more explicit by setting
  $$ {w}_{res} = \mathrm{c}{K}_2, $$
  where *c* > 0 describes the relative contribution of *w*_*res*_; Eq. (12) then becomes
  13 $$ b=\frac{1}{c^2+1}. $$
  A relatively small residual *c* (and thus, c^2^) would explain why values of *b* are often close to 1. Furthermore, since *w*_*res*_ is fixed and *K*_*2*_ is proportional to *σ* (cf. Eq. (9)), *c* is inversely proportional to *σ*, which may explain why accuracy of correlation perception improves when dot clouds have smaller standard deviations (Cleveland et al., 1982).^9^ The larger standard deviations for uniform distributions might likewise account for the (nonsignificantly) larger biases found in Experiment 4: the ratio of .29/.2 for the relative sizes of the standard deviation would predict *b* = .95, close to the observed value of .94.
  Note that these developments do not rely on the way that information is represented; instead of corresponding to positions in the image, dimensions *x* and *y* might correspond to values in a more abstract parameter space. This could explain the existence of similar laws when other graphical representations are used (Harrison et al., 2014; Rensink, 2014, 2015).
iii) *Information entropy*. The assumption that perceived correlation depends on the width of the probability distribution can account for the empirical results found here, as well as those of several other studies. But why would human vision be concerned with this particular quantity (cf. Marr, 1982)? And given that the square of the width (cf. Eq. (8)) is an accurate estimator of correlation, why would the visual system instead use its logarithm? As a possible answer, an *entropy theory* is proposed here: observers can perceive the information entropy in a scene, with this quantity then used as a proxy for correlation.
  Information entropy (Shannon entropy) can be defined as *H* = − ∑*p*(*x*_*i*_, *y*_*i*_)ln *p*(*x*_*i*_, *y*_*i*_) (see, e.g., Lemons, 2013). This is an inherently statistical quantity, reflecting the number of possible configurations of a given probability distribution. Physical entropy—the physical instantiation of *H*—is useful for describing many natural structures (see e.g., Ben-Naim, 2008; Lemons 2013; Weber, Depew, & Smith, 1988). Information entropy could likewise be a useful descriptor of visual structure. In computer vision systems measures based on entropy have been used to assess statistical structure in images of real-world scenes and textures (e.g., Chang, Du, Wang, Guo, & Thouin, 2006; Zhu, Wu, & Mumford, 1998). In human vision, it has been suggested that eye movements are guided by the perception of contrast entropy, enabling the greatest amount of information gained at each fixation (Raj, Geisler, Frazor, & Bovik, 2005; Renninger, Verghese, & Coughlan, 2007). A variant of this quantity—the number of bits needed to encode an image using a subset of wavelets—has also been considered as a possible measure of the visual clutter in a scene (Rosenholz, Li, & Nakano, 2007).
  In general, entropy is difficult to estimate (Archer, Park, & Pillow, 2014). For a bivariate gaussian distribution, however, the situation can be simplified. The differential entropy^10^ of the corresponding probability density function (Eq. 4) is
  14 $$ H(r)= \ln \left(2\pi e{\sigma}_x{\sigma}_y\sqrt{1-{r}^2}\right) $$
  (see e.g., Gokhale, Ahmed, & Res, 1989); for a distribution of *n* dots, this simply becomes
  15 $$ H(r)=n \ln \left(2\pi e{\sigma}_x{\sigma}_y\sqrt{1-{r}^2}\right) $$
  Meanwhile, the isofraction ellipse *q*(*x*,*y*) = *K*_1_^2^ for the dot density function *nf*(*x*,*y*) has an area *A* given by (e.g., Johnson, 2015)
  16 $$ A(r)=2\pi {\sigma}_x{\sigma}_y{K}_1^2\sqrt{1-{r}^2} $$
  allowing Eq. (15) to take the form
  17 $$ H(r)=n \ln \left(Ae/{K}_1^2\right)=n \ln (A)+n \ln \left(e/{K}_1^2\right) $$
  Given that the methods used here involve only differences in *g*(*r*), entropy theory implies
  18 $$ g\left({r}_1\right)-g\left({r}_2\right)=G\hbox{'}\left(H\left({r}_1\right)-H\left({r}_2\right)\right), $$
  or equivalently,
  19 $$ g(r)=G\hbox{'}\left[H(r)+h\hbox{'}\right], $$
  where *G*' and *h*' are real-valued constants. Substituting Eq. (17) into Eq. (19) leads to
  20 $$ g(r)=G\left[ \ln \left(A(r)\right)+ \ln \left(e/{K}_1^2\right)+h\hbox{'}\right]. $$
  where *G* = *nG*'.
  The issue now is to determine the area *A* of the isofraction ellipse. For geometric structures, the perceived area of an ellipse is the product of separate one-dimensional measurements (Morgan, 2005); the existence of capacity limitations for ensemble coding (Attarha & Moore, 2015) would suggest a similar situation here. If only one ensemble descriptor can be determined at a time, *A*(*r*) might be approximated by the product of the width *w*(*r*) of the ellipse and some fixed value *D* representing its length; Eq. (20) then becomes
  21 $$ g(r)\approx G\left[ \ln \left(w(r)\right)+ \ln (D)+ \ln \left(e/{K}_1^2\right)+h\hbox{'}\right]. $$
  Setting *h* = *h* ' + ln(*D*) + ln(*e*/*K*_1_^2^) yields Eq. (7), which—as shown in the previous section—becomes Eq. (2) when σ_x_ and σ_y_ are equal. This approximates the exact formula for entropy (Eq. (20)) fairly well (Fig. 13).

**Fig. 13:**
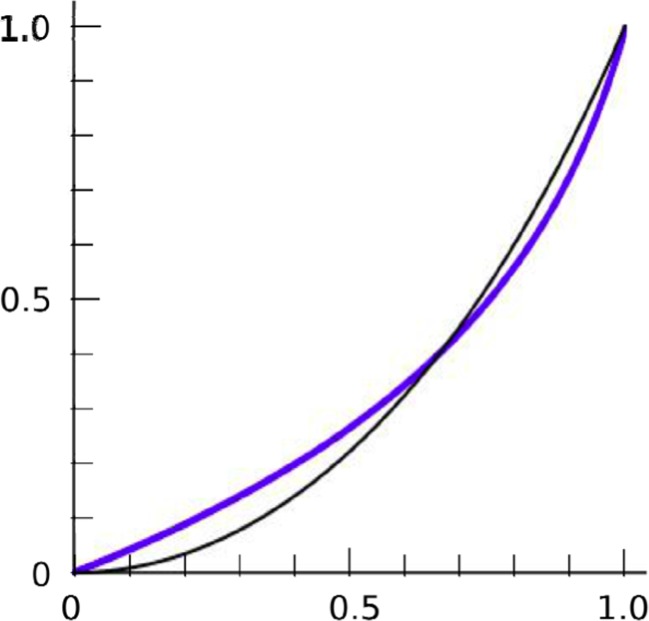
Approximation of entropy. Fechner's law (Eq. (2); thicker line) vs. exact formula based on entropy (Eq. (20); thinner line). Both have been adjusted to have best fits to the data of Experiment 1. As is evident, Fechner’s law is slightly higher at low correlations and slightly lower at high ones. But the two curves match each other reasonably well: average RMSE over the range 0 ≤ *r* ≤ 1 is only 0.04
  To show the linkage between discrimination and estimation, the derivative of Eq. (2) yields
  22 $$ \varDelta g(r)/\varDelta r\approx dg/dr=-b/\left[\left(1-br\right) \ln \left(1-b\right)\right]. $$
  Because of the close approximation of Eq. (2) to the actual entropy, any change Δ*g* is reasonably proportional to the associated change Δ*H*. And because Δ*H* is a measure of information gain (see e.g., Lemons, 2013), all Δ*g*'s of equal size involve about the same number of bits. In this situation, then, the Fechner assumption that each JND corresponds to the same difference in subjective experience Δ*g* takes the weaker form that each JND corresponds to the same number of perceived bits *I*_*75*_ (where the subscript denotes 75 % discrimination accuracy^11^). Letting *JND*(*r*) denote the value of Δ*r* that corresponds to this number, Eq. (22) becomes
  23 $$ JND(r)\approx -{I}_{75}\left(1-br\right) \ln \left(1-b\right)/\left(-b\right)=-{I}_{75} \ln \left(1-b\right)\left(1/b-r\right), $$
  which then becomes Eq. (1) by setting *k* = − *I*_75_ ln(1 − *b*). (Note that since *I*_*75*_ depends on the particular representation and number *n* of data points used, if these are constant, *k* will be proportional to -*ln*(*1*-*b*).^12^) The finding that discrimination and estimation lead to the same estimates of *b* shows that this weaker form of the Fechner assumption holds fairly well. It is worth noting that the resulting logarithmic relationship between physical and perceived quantities is relatively rare, since most such relationships involve power laws rather than logarithms (Billock & Tsou, 2011).
  Interestingly, the above treatment also goes through if *g*(*r*) were proportional to *entropy density*, an intensive property that describes the amount of entropy per unit area (with this area being, e.g., that subtended by the scatterplot axes). Because this differs formally from information entropy only by a multiplicative constant, the calibration done to set *g*(0) = 0 and *g*(1) = 1 would result in no formal difference in *g*(*r*), and thus, no difference in performance.
iv) *Unequal standard deviations*. Much of the development above assumes that the standard deviations of both dimensions are equal (i.e., *κ* = 1). When this is not the case, two limiting cases can be singled out. First, when *σ*_y_ << *σ*_x_ (*κ* → 0), Eq. (6) becomes
  24 $$ w(r)=2\sqrt{2}{K}_1{\sigma}_y\sqrt{1-{r}^2}. $$
  Second, when *σ*_y_ >> *σ*_x_ (*κ* → ∞), Eq. (6) becomes
  25 $$ w(r)=2\sqrt{2}{K}_1{\sigma}_x\sqrt{1-{r}^2}. $$
  In both cases, a similar development as above yields
  26 $$ g(r)=\frac{ \ln \left(1-b{r}^2\right)}{ \ln \left(1-b\right)}, $$
  where *b* is as before, but with ${K}_2=2\sqrt{2}{K}_1{\sigma}_y$ when *σ*_y_ << *σ*_x_, and ${K}_2=2\sqrt{2}{K}_1{\sigma}_x$ when *σ*_y_ >> *σ*_x_. A development parallel to that for Eq. (23) then yields the JND curve
  27 $$ JND(r)=k\hbox{'}\left(1/br-r\right). $$
  Since *A*(*r*) is always proportional to $\sqrt{1-{r}^2}$ (Eq. 16), Eqs. (24) and (25) have an exact match with *A*(*r*) under these conditions. Consequently, Eq. (21) should be a reasonable approximation for most choices of σ_x_ and σ_y_.

### Comparison with previous work

The basic stage of correlation perception in scatterplots has been the focus of many studies over the years. Relatively few, however, examined discrimination. Pollack (1960) and Doherty et al. (2007) characterized this in terms of *d*' (signal to noise ratio), finding performance to be better at high correlations; similar results were obtained by Li, Martens, and van Wijk (2010). These are all consistent with the results found here. Indeed, transforming the *d*' measures of Experiment 2 of Doherty et al. (2007) into JNDs (these quantities being inversely related) yields a highly linear behavior (*R* ^2^ = 0.972) that obeys Eq. (1), with variability *k* ≈ 0.17 and bias *b* ≈ 0.90, values not far from those found here. Harrison et al. (2014) found that Eq. (1) held for several kinds of graphical representation (e.g., line plots), again with parameters broadly similar to those found here.

The more intensively-studied aspect of correlation perception, however, is magnitude estimation. (For reviews, see Boynton, 2000; Doherty et al., 2007; Konarski, 2005). Several studies have proposed particular equations for the relationship of perceived to physical correlation. Table 1 shows the most popular ones (and a few variants) and their fits to the estimation data obtained here. These proposals are grouped according to the number of free parameters they contain:

**Table 1:**
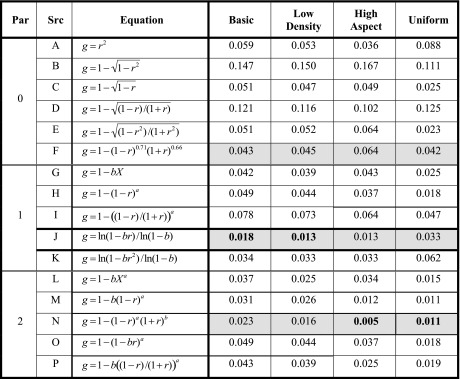
Fits of perceived correlation

RMSE of various proposals for perceived correlation *g*(*r*); fits are to the seven levels of perceived correlation measured here. Gray squares indicate best overall fit for each number of free parameters; numbers in bold are the best fits for each condition

Parameters (Par): The number of free parameters in the equation. Source (Src): (A) Variance (Pollack, 1960). (B) Coefficient of alienation *w*(*r*) of Jennings, Amabile, and Ross (1982); based on area of isopleths. (C) Modified version of *w*(*r*), based on width rather than area. (D) *g*(*r*) of Cleveland et al., 1982; based on the ratio of minor to major axes. (E) Version of *g*(*r*) modified b*y* Meyer et al. (1997). (F) Double-power law of note 15 of Cleveland et al. (1982), with suggested constants *a* = 0.71 and *b* = 0.66. (G) Linear function of average absolute perpendicular distance *X* from the regression line (Meyer et al., 1997). (H) Power law for distance from *r* = *1*, with exponent *a* (best fit *a* ≈ 0.5 for most conditions). (I) Power law for ratio of minor to major axes (Boynton, 2000); best fit is *a* ≈ *0.38* for most conditions. (J) Fechner's law (Eq. (2)). (K) Logarithmic function of *r* ^2^ instead of *r*—the entropy based on the true area of the isopleth ellipse. (L) Power law for average absolute perpendicular distance *X*, with two parameters (Meyer et al., 1997). (M) Power law for distance from *r* = *1*, with two free parameters. (N) Double-power law of Cleveland et al. (1982; note 15). (O) Power law for *u*=*1*-*br*, with exponent *a* (best fit *a* ≈ 0.5, *b* = *1* for all conditions). (P) Modified power law for ratio of minor to major axes; variant of the measure proposed in Boynton (2000)

i) *Free parameters* = *0*. These are simply functions of correlation *r*. As is evident from Table 1, average RMSE is about 0.08. Note that *g* = *r* ^2^ (row A) is not the best model for most conditions, indicating that the underlying quantity is unlikely to be simple variance, as was sometimes suggested (e.g., Pollack, 1960; Strahan & Hansen, 1978). More generally, the relatively poor fit for these equations supports the proposal that—even for simple bivariate gaussian distributions—perceived correlation depends upon more than *r* alone (Boynton, 2000; Lane et al., 1985).
ii) *Free parameters* = *1*. When a single free parameter is allowed, the best fit is with Eq. (2)—row (J); RMSE here is less than half that of the other models with one free parameter, at least for gaussian distributions. The fit remains good for uniform conditions as well, although the somewhat higher RMSE here suggests that gaussian distributions may in some sense be the more natural ones.^13^ Note that the fit is better than for the average perpendicular distance X (row G); since X is essentially proportional to the width of the distribution, the somewhat better fit with Eq. (2) would seem to be due to the use of the logarithm and the presence of a residual. The fit also tends to be better than for a power of the distance from *r* = *1* (row H), or of the ratio of the width of the isofraction ellipse to its length (row I). Note that power laws—which describe the perceived magnitude of most perceptual properties (see e.g., Billock & Tsou, 2011; Ross, 1997)—do not generally fit much better than equations based only on *r*. Importantly, the fit with Eq. (2) is also better than for the logarithm of 1-*br* ^2^, (row K), the accurate equation for entropy.
iii) *Free parameters* = *2*. Table 1 shows that the double-power law of Cleveland et al. (row M) has the best fit of any two-parameter proposal. This is not entirely a surprise: all things being equal, fit should be better for equations with more free parameters. However, Eq. (2) still provides the best global fit for the basic and low-density conditions, as well as the second-best fit overall, despite having only one free parameter (bias).

All things considered, then, Fechner’s Law (Eq. (2)) fits the data at least as well as any other proposal to date. Moreover, it also shows a systematic link between discrimination and estimation, and points to a mechanism that connects it with the rest of visual perception. Note that in this formulation, perceived correlation—especially for gaussian distributions—essentially involves just one parameter, bias *b*, which summarizes the effects of all the factors that influence correlation perception, essentially acting as a modulator.

Although care must be taken when comparing the results of different experiments, a few tendencies are apparent. For the low-density condition (Experiment 2), *k* is considerably higher (0.30 [0.26, 0.35], vs. 0.21 [0.17, 0.24]; *t*(38) = 3.29; *p* = .002), a phenomenon also found by Doherty et al. (2007) and Rensink (2014). This likely reflects the greater sampling noise due to the smaller number of dots. Bias was not noticeably affected, consistent with the pilot studies reported by Bobko and Karren (1979). Although it is sometimes stated that perceived magnitude—and therefore bias—is affected by density (e.g., Boynton, 2000), those studies manipulated density by changing the standard deviation of the dot cloud; when density is manipulated by changing the number of dots present, effects are much weaker (Lauer & Post, 1989; Rensink, 2014). Finally, in the uniform condition (Experiment 4), bias tended to be somewhat higher; if so, it may be either because the uniform distributions did not match the gaussian structure assumed by the perceptual systems involved (Utochkin, 2015; Utochkin & Tiurnia, 2014), or simply because of the greater standard deviation.

### Applications to visualization

The view of correlation perception put forward here has several implications for information visualization. To begin with, the relatively simple nature of Eqs. (1) and (2) suggests an interesting application to the evaluation of designs. Visualization designs are typically evaluated using user studies, which can often be quite time-consuming (Carpendale, 2008). The results here, however, suggest that some aspects of this process—at least for the visualization of correlation using scatterplots—could be done in a considerably faster way. For example, if the datasets being visualized have near-gaussian distributions, and if each level of correlation is equally likely to be encountered, precision can be characterized by the scatter *S*, defined as the average JND over the range 0 ≤ *r* ≤ 1; this corresponds to the area under the curve of Eq. (1), or equivalently for this situation, its value at the midpoint *r* = 0.5:

28 $$ S=k\left(\frac{1}{b}-\frac{1}{2}\right),\kern0.5em 0<k,b<1. $$

Accuracy can likewise be characterized as the average (under)estimation error *E*, corresponding to the average difference between g(*r*) and *r*. Via Eq. (2), this takes the form:

29 $$ E=\frac{1}{b}-\frac{1}{2}+\frac{1}{ \ln \left(1-b\right)},\kern0.5em 0<b<1. $$

Using these formulae, any scatterplot design (e.g., one with a particular size or color of dots) can be rated in terms of precision and accuracy, at least for the visualization of correlation in near-gaussian distributions: all that is needed are the parameters *k* and *b*.^14^ Different dot colors, sizes, etc. could likewise be compared. And even if Eqs. (28) and (29) turn out to be valid only for near-gaussian distributions, any results found using these (for different colors, say) might still be applicable for other kinds of distributions.

An important issue is to what extent such evaluation could be sped up. In the experiments here, parameters *k* and *b* were measured using a fairly large set of base correlations. This was done to enable the shape of the performance curves to be mapped out in detail. But if conditions are similar to those tested here, *k* and *b* might be measured using far fewer tests. To examine the feasibility of this, the discrimination data in Experiments 1–4 were reanalyzed using a smaller number of base correlations: either {0.3, 0.5, 0.7, 0.9}, {0.3, 0.6, 0.9}, or {0.3, 0.9}, with JNDs from above. In addition, magnitude estimates were made using 3 subdivisions (corresponding to the first two stages of the method used here), and a single (initial) subdivision.^15^ As seen from Table 2, estimates were quite robust, remaining largely the same. The only exceptions were significantly higher values for *b*_disc_ when two base correlations were used on distributions with low densities or high aspect ratios, and a (nonsignificant) trend toward higher values for *b*_est_ when just one subdivision was used on uniform distributions.

**Table 2:**
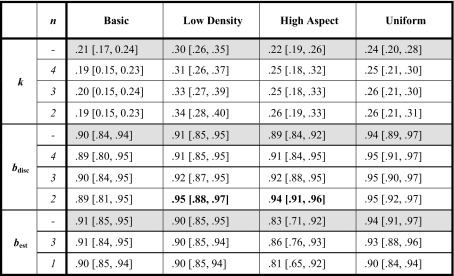
Estimates using different numbers of sampling points (*n*). Upper row for each parameter (in gray) corresponds to the estimates given in the main text

As is apparent, reducing the number of points does not cause estimates to deteriorate greatly. The two significant variations in measurements are in bold; these were for 2-point estimates of *b*_disc_, for low densities and high aspect ratios, which were significantly higher than those for large numbers of sampling points. Square brackets indicate 95 % CIs. To reduce clutter, leading zeroes have been omitted

Based on these results, a simple method of assessing of *k* and *b* can be suggested: measure JND from above for three base correlations (e.g., 0.3, 0.6, and 0.9), and fit the results to Eq. (1). Owing to the link between discrimination and perceived magnitude, no bisection is needed, although if the Fechner assumption is to be tested, a small number of subdivisions (3, or possibly even 1) should suffice. For maximal sensitivity, a within-observer design could be used, with each observer tested on the same set of (counterbalanced) designs.

The developments in this paper also have applications at a more general level. For example, the proposal that correlation is based on probability distributions over an abstract parameter space implies that the values of data points need not be conveyed by spatial position—they could instead be represented by other properties, such as color or orientation (cf. Fig. 11). Given that the perception of correlation in such representations is similar to that found in scatterplots (Rensink, 2014, 2015), and given that such visualizations could take up less space (Fig. 11), there may be practical advantages to their use.

More generally yet, the developments here show that our understanding of visualization can be improved not only via knowledge of the *mechanisms* underlying human perception and cognition (e.g., Card et al., 1999; Ware, 2012), but also via the *methodologies* used to obtain that knowledge. Indeed, developing this approach in a more thorough and systematic way may even result in a science of visualization for some aspects of this domain, an area of research that could connect with several parts of psychology—in particular, vision science (Rensink, 2014),

### Applications to vision science

This study has shown that for gaussian and uniform distributions, the perception of correlation in scatterplots can be described by a pair of simple laws: a linear one for discrimination and a logarithmic one for magnitude. These laws appear to derive from the width of inferred probability distributions, which in turn may reflect the perception of entropy in the image. If so, there would be several important implications for our understanding of the mechanisms underlying visual perception.

The proposal of inferred probability distributions suggests that the mechanisms involved in correlation perception may be related to those underlying ensemble coding (see Alvarez, 2011; Haberman & Whitney, 2012). Early studies of ensemble coding focused on simple scalar properties (estimators), such as mean size (Ariely, 2001), orientation (Dakin & Watt, 1997), and center of mass (Alvarez & Oliva, 2008; Drew, Chubb, & Sperling, 2010). However, it has become increasingly clear that these mechanisms can also respond to the *shape* of the underlying probability distributions, at least over one-dimensional spaces (Chetverikov, Campana, & Kristjansson, 2016; Utochkin, 2015). The results here suggest that the shape of *two*-dimensional distributions can also be determined this way, with this shape serving as the basis of entropy estimation. (Note that this shape need not be used exclusively for this purpose; it might also serve as the basis for other things, such as categorization.) The results also suggest that—similar to the limits on the number of ensemble properties can be determined at a time (Brand, Oriet, & Tottenham, 2012)—limits exist on the number of properties that can be concurrently determined about the shape of such distributions.

Another connection involves the proposal that the goal of the mechanism outlined here is the perception of the entropy in an image. Statistical structure has long been thought to play a critical role in the visual perception of scenes (see e.g., Geisler, 2008; Haberman & Whitney, 2012; Olshausen & Field, 1996; Rensink, 2000), with several statistical quantities apparently perceived quite rapidly (e.g., Fei-Fei, Iyer, Koch, & Perona, 2007; Oliva & Torralba, 2006).^16^ Given that entropy is an important statistical structure, it is reasonable to suppose that it too might be such a quantity. Among other things, entropy has been suggested as an important quantity in the guidance of eye movements (Raj et al., 2005; Renninger et al., 2007) as well as a possible measure of clutter in a scene (Rosenholz et al., 2007). The proposal here supports suggestions of this kind; indeed, similar—or even the same—mechanisms may be involved. And if correlation perception does indeed reflect a form of entropy perception, it would indicate that entropy is not only *used* in perceptual processing, but can be *accessed* by higher-level mechanisms. The use of scatterplots to visually convey structure would then be an interesting example of this ability being harnessed for practical purposes.

In any event, the view proposed here consolidates much of the work on the perception of correlation in scatterplots over the past several decades. In particular, it can account for several key aspects of this process:

i. The logarithmic form of perceived correlation *g*(*r*).
ii. The linear form of JND(*r*), possibly via an intensive quantity (entropy density).
iii. The linkage between these two quantities (Fechner assumption).
iv. The relative indifference of these to the presence of individual outliers.
v. The greater accuracy (lower bias *b*) when the standard deviation of the cloud is smaller.
vi. The considerable indifference of *b* to the density of dots in a scatterplot.
vii. The invariance of the above in regards to different ways of representing information (e.g., via position or color).

Several predictions also follow:

i. The two-dimensional shape of probability distributions can be determined in ensemble coding (although only a single aspect of it may be accessible at any one time).
ii. Perceived correlation *g*(*r*) is a logarithmic function of *v*=*1*-*br* ^*2*^ in situations where the two dimensions have markedly unequal standard deviations.
iii. If the Fechner assumption holds, JND(*r*) will be proportional to (*1*/*br* - *r*) under such conditions.
iv. For a given property to represent information, a given number of data elements, and a given way to measure JND, variability *k* will be proportional to -*ln*(*1*-*b*).
v. The above aspects and predictions will hold to the extent that the property used to represent information obeys an *isometric constraint*—equal perceptual distances map to equal distances in numerical (value) space. The properties for which this is possible will likely be related to the basic features of visual perception (e.g., Treisman, 1988).
vi. Different ways of representating correlation can be compared without any great loss in performance—e.g., the correlation in an augmented stripplot of the type shown in Fig. 11 could be accurately matched against that in a scatterplot, or JNDs would be much the same when measured using scatterplots with different densities or aspect ratios.^17^
vii. It should be possible to adapt to correlations conveyed by different graphical representations.

### Future directions

Although the view put forward here can explain much of correlation perception, many issues still remain to be investigated. For example, what happens at transitions between positive and negative correlations? When base correlations less than 0.2–0.3 are removed, *post*-*hoc* analysis shows that behavior for uniform distributions is similar to that of the basic condition (Experiment 4). This suggests that a transition of some kind may exist there, in which the proxy for correlation at high values (entropy) is replaced at low ones by a different one (e.g., density), possibly due to the latter quantity supporting a stronger signal. If so, distinct "zones" for high and low correlations may exist, with interesting effects at their transition points.^18^

Another important set of issues concerns the nature of the data distributions themselves. Although the laws here apply fairly well to at least some non-gaussian distributions, it is not clear how far this goes. Distributions with the same means, standard deviations, and correlations can vary considerably in their structure (Anscombe, 1973). It would be useful to know how far the approach developed here would apply. Another issue is the extent to which a second, irrelevant distribution can affect performance (cf. Konarski, 2005; Lewandowsky & Spence, 1989; Wainer & Thissen, 1979). More generally, it may be worth looking at the extent to which multiple distributions can be separated out, based on the two-dimensional shape of the probability distribution; this might be investigated by an adaptation of current approaches to segmenting probability distributions (e.g.. Cohen et al., 2008; Feldman, Singh, & Froyen, 2013; Utochkin, 2015). A related issue is whether the gaussian is a natural distribution for the processes involved, as appears to be the case for ensemble coding (Alvarez, 2011; Utochkin & Tiurnia, 2014). Other issues in this vein include the extent to which nonlinear correlations can be perceived, and whether information entropy could also account for the perception of correlation in higher-dimensional datasets.

A somewhat different set of questions concerns the effectiveness of display factors such as the size and shape of the dot cloud, or the size and shape of its dots to convey correlation (cf. Cleveland & McGill, 1984a). If gaussian distributions are used to test these, the evaluation procedure suggested above could be readily applied and the values of *k* and *b* measured; once this has been done, Eqs. (28) and (29) could provide quantitative measures of precision and accuracy for each design parameter. Results of this kind would not only be of practical importance, but might also cast further light on the nature of the perceptual mechanisms involved. Indeed, investigation into the kinds of properties that give rise to laws similar to those found here could provide a new, independent source of insight into the nature of the visual features believed to support the early stages of perception.

In this context it is worth mentioning that the aspect of correlation perception investigated here is its basic stage—i.e., the part that is carried out rapidly and intuitively by most observers. The finding that this is largely complete within 100–150 ms (Rensink, 2014) suggests a similarity to the initial stage of scene perception, where processes are spatially parallel and act rapidly, typically within a few hundred milliseconds (see e.g., Rensink, 2000). If these stages turn out to be identical, some interesting implications follow. For example, the estimates used in the probability distributions could be properties of *proto*-*objects*—localized structures believed to be created early in visual processing (Rensink & Enns, 1995). If so, correlation perception would have interesting connections to visual search and clutter perception, both of which appear to be based on measurements derived from proto-objects rather than raw pixels in the image (e.g., Rensink & Enns, 1995; Yu, Samaras, & Zelinsky, 2014).

And just as scene perception has an attentional stage that depends on the knowledge of the observer, so does correlation perception have a subsequent stage that supports more sophisticated operations, such as the selection of particular data points (Freedman & Smith). Although the extent to which this stage involves attention is not yet clear, it does appear to require deliberation and is aided by expertise (e.g., Lewandowsky & Spence, 1989). As such, many of the same processes may be involved, further supporting the proposal of a deep connection between vision and visualization (Rensink, 2014).

Clearly, more can be perceived in a scatterplot than just correlation: many other kinds of visual structure are possible. Possible candidates include not only clusters and outliers, but also such things as the convexity, skinniness, or clumpiness of the dot cloud itself (Wilkinson, Ananad, & Grossman, 2005; Wilkinson & Wills, 2008). Techniques analogous to those described here might be developed to explore such possibilities.

Finally, it may be worth emphasizing that research issues in vision science and information visualization are often interlinked: the design of a visualization can often be aided by knowledge of the underlying perceptual mechanisms, while careful investigation into its operation can shed new light on the nature of these mechanisms (Rensink, 2014). This study is one example of how the latter could be done. But examples also exist for other aspects of visualization, such as the perception of average value (e.g., Gleicher, Correll, Nothelfer, & Franconeri, 2013; Legge, Gu, & Lubker, 1989) and the perception of structure in graphs (Cleveland & McGill, 1984b). More generally, the graphical representations used to display data can form a useful class of stimuli for research into human perception and cognition. It is sometimes believed that artifacts have arbitrary structure, and as such are irrelevant for the study of human perception and cognition. But although humans did not evolve to work with artifacts, artifacts in common use essentially evolved to work with us. Representations such as scatterplots are survivors of considerable competition; there are likely good reasons why they remain in use. Finding those reasons may therefore not only help us better understand the kinds of visualizations that have been or could be developed, but may also help give us new insights into the nature of our perceptual and cognitive systems.

## Footnotes

1 All but one of these had a near-zero value of *b* in the bisection task; these observers, however, had the same value of *b* as others in the discrimination task. This suggests that these observers did not use the same quantity for discrimination and magnitude estimation (or use the same quantity for magnitude estimation as the other observers). It is unclear what this property might have been. However, a trimming of 10–20 % of the observers has long been done in studies of correlation perception in scatterplots (e.g., Cleveland et al., 1982), supporting the possibility that alternate strategies are used by subsets of the general populace.

2 A within-observer pilot study compared four initial differences (0.0, 0.1, 0.2, and 0.3), and found no significant effect on JND. The value of 0.1 was chosen because it tended to require a minimum number of trials on average.

3 Variances were calculated using a denominator of 7 for the variance within each window, and 2 for the variance between windows.

4 For these, the value used was the average of the last 24 trials.

5 When data from the two lowest base correlations (*r* = 0.0 and 0.1) were removed from the analysis, slopes became much more alike (*t*(19) = 0.75; *p* = .46), as did intercepts (*t*(19) = 1.07; *p* = .30), with average *k* = 0.24 [0.20, 0.28]. The divergence may therefore have been caused by the squarish shape of the dot clouds at low levels of correlation. These two conditions are therefore omitted in the subsequent analysis.

6 In contrast, properties such as area or length are *extensive*, behaving in a rather different way (see, e.g., Carnap, 1966). For example, the area of a given region is the *sum* of the areas of its component sub-regions.

7 The correlation *r* is technically the population parameter ρ. In the work here, however, *r* is set to the same value in all instances, and so is used here to minimize notational switching.

8 Isofraction lines are a special type of isopleth (lines of equal probability). By definition, they have the property that the same fraction (determined by K_1_) can specify an ellipse independent of any particular value of *r* or *n*.

9 Fitting the data in Fig. 2 of Cleveland et al. to eq. (2) yields *b* values of .91, .89, .94, and .96 for increasing sizes of the dot cloud. Taking the reference value to be *b* = .89 (size 2 in Cleveland et al), predicted *b* values are .84, (.89), .94, and .97 respectively, a good match to most of the data. Note that size 2 in Cleveland et al. is roughly that of the basic condition here; the similar values of *b* indicate a degree of consistency across studies.

10 Although much the same as entropy for the discrete case, differential entropy—based on a probability density function rather than an array of discrete probabilities—can be negative in some situations (e.g., as *r* goes to 1 in eq. (9)). However, it is a good approximation here, with the fixed residual width *w*_res_ preventing the argument of the logarithm from becoming arbitrarily small. Moreover, the approach here relies on *differences* in differential entropy, which does not have such problems (see e.g., Norwich, 1987).

11 The value of a threshold depends somewhat on the method used to measure it (see e.g., Chong & Treisman, 2005; Treutwein, 1995). Thus, *I*_75_—and therefore, *k*—may differ slightly for different methods. But it will be constant when the same method is used throughout.

12 This may explain why variability in the uniform distribution (Experiment 4) tends to be higher than in the basic condition (Experiment 1). If *b* is greater in the uniform distribution (*b* = 0.94 [0.89, 0.97] vs. *b* = 0.91 [0.85, 0.95], then *k* = 0.21 * ln(1-0.94)/ln(1-0.91) = 0.245, quite close to the *k* = 0.24 observed. Given that these trends were not found to be significant in the present study, another study with more power would be needed before conclusively accepting or rejecting this proposal.

13 This pattern can also be seen in terms of CIs: For the three gaussian distributions, eq. (2) falls within the 95 % CIs for all values tested. For the uniform distribution, this did not occur for one value (the highest); distance beyond the 95 % CI here was 0.01.

14 Clearly, such measures may not be relevant for other uses of scatterplots, such as the visualization of clusters or outliers. They also do not capture aspects such as the aesthetics of the display.

15 Consistent with the approach used in the main analysis, values of *b* or *k* were dropped if they were outside the range of 2.5 standard deviations. However, in this case the associated observers were not replaced.

16 Statistical representations in vision can use two different kinds of referencing: one based on sets of *positions*, the other on sets of *items*. Position-based referencing is the basis of representations such as texture (e.g., Bergen & Adelson, 1988); item-based referencing is the basis of ensemble coding. For a scatterplot, correlation stems from the positions *derived* from a set of items (dots); the process does not necessarily use the position of each dot to *reference* it.

17 Preliminary experiments appear to support this.

18 Such a phenomenon has also been found in pilot experiments on parallel co-ordinate plots (Lane Harrison, personal communication; Kyle Melnick, personal communcation).
